# Supplementary material for: MEK inhibition reduced vascular tumor growth and coagulopathy in a mouse model with hyperactive GNAQ
Source: Nat Commun. 2023 Apr 6;14:1929. doi: 10.1038/s41467-023-37516-7 (PMC10079932; doi:10.1038/s41467-023-37516-7)
Supplement: Supplementary file 1 — Supplementary Information [file 41467_2023_37516_MOESM1_ESM.pdf]

Supplementary Information

Title: MEK inhibition reduced vascular tumor growth and coagulopathy in a mouse model with hyperactive GNAQ

Author list: Sandra Schrenk[1,2], Lindsay J Bischoff[1,3], Jillian Goines[1], Yuqi Cai[1], Shruti Vemaraju[4,5], Yoshinobu Odaka[5,6], Samantha R Good[1], Joseph S Palumbo[2,7], Sara Szabo[8], Damien Reynaud[1,2], Catherine D Van Raamsdonk[9], Richard A Lang[4,5,10,11], Elisa Boscolo[1,2]*

Content:
Supplementary Tables 1-5
Supplemental Figure 1-19 and Supplementary Figure legends

Other Supplementary Materials for this manuscript include the following:

Supplementary Movies 1-12

Source Data

Supplementary Tables

Supplementary Table 1. Table of mendelian ratios of embryos with constitutive ubiquitous expression of *GNAQ*^{Q209L}

Age of pups/embryos	Expected (Percentage)		Observed		Chi-square value	p-value
	CMV-Cre	CMV-Cre; GNAQ^{Q209L}	CMV-Cre	CMV-Cre; GNAQ^{Q209L}		
**Postnatal**	50%	50%	13	0	13	0.0003***
**E13.5**	50%	50%	7	0	7	0.0082**
**E8.5**	50%	50%	6	1	2.667	0.1025

Supplementary Table 2. Genotyping primers

	Forward (5’-3’)	Reverse (5’-3’)
**GNAQ Q209L**	TAGCTGACCCTGCCTACCTG	CCTCCATTCGGTTCTCATTG
**Cdh5-Cre**	GCGGTCTGGCAGTAAAAACTATC	GTGAAACAGCATTGCTGTCACTT
**Pdgfb-Cre**	CCAGCCGCCGTCGCAACT	GCCGCCGGGATCACTCTCTCG
**CMV-Cre**	ACCAGCCAGCTATCAACTCG	TTACATTGGTCCAGCCACC
**tdTomato**	GGCATTAAAGCAGCGTATCC	CTGTTCCTGTACGGCATGG

Supplementary Table 3. List of antibodies

Host Species	Species Reactivity	Antigen	Conjugate	Company	Catalog number	Clone	Concentration	Application
Rabbit	Mouse	CD31	N/A	Cell Signaling	77699	D8V9E	0.062μg/mL	IHC-P
Rabbit	Human, Mouse	Ki67	A488	Cell Signaling	11882	D3B5	2μg/mL	IHC-P (IF)
Rabbit	Human	ERG	A647	Abcam	ab196149	EPR3864	5μg/mL	IHC-P (IF)
Rabbit	Human, Mouse	pERK 1/2 (Phospho-p44/42 MAPK Thr202/Tyr204)	N/A	Cell Signaling	9101	polyclonal	5μg/mL	IHC-P (IF), WB
Mouse	Human, Mouse	ERK 1/2	N/A	Cell Signaling	4696	L34F12	0.5μg/mL	WB
Rat	Mouse	CD31	N/A	BD Biosciences	550274	MEC 13.3	0.075μg/mL	IF-Whole-mount
Rat	Mouse	TER119	A647	BioLegend	116218	TER-119	5μg/mL	IF-Whole-mount, IHC-Fr (IF)
Rat	Mouse	CD41	PE	BD Biosciences	558040	MWReg30	1μg/mL	IF-Whole-mount, IHC-Fr (IF)
Rabbit	Human, Mouse	CD42b	N/A	Abcam	183345	SP219	0.3μg/ml	IF-Whole-mount, IHC-Fr (IF)
Mouse	Human	VE-cadherin	A647	BD Biosciences	561567	55-7H1	0.5μg/mL	ICC-IF
Rabbit	Human, Mouse	GNAQ		Cell Signaling	14373	D5V1B	0.05μg/mL	WB
Mouse	Human, Mouse	VE-cadherin	N/A	Santa Cruz	sc-9989	F-8	0.5μg/mL	WB
Rabbit	Human, Mouse	pAKT (Ser473)	N/A	Cell Signaling	4060	D9E	0.5μg/mL	WB
Mouse	Human, Mouse	AKT	N/A	Cell Signaling	2920	40D4	0.2μg/mL	WB
Goat	Human	Angiopoietin-2	N/A	R&D Systems	AF-623	polyclonal	1μg/mL	WB
Mouse	Human, Mouse	GAPDH	N/A	Millipore	MAB374	6C5	0.2μg/mL	WB
N/A	N/A	UEA I	DyLight™ 649	Vector Laboratories	DL-1068-1	N/A	20μg/mL	IHC-IF
**Secondary antibodies**								
Goat	Anti-rabbit		A594	Invitrogen	A32740		8μg/mL	IHC-IF
Goat	Anti-rabbit		biotinylated	Vector laboratories	BA-1000		7.5μg/mL	IHC-IF
Goat	Anti-rat		biotinylated	Vector laboratories	BA-9401		7.5μg/mL	IHC-IF
			Streptavidin Peroxidase (HRP)	Vector laboratories	SA-5004-1		5μg/mL	IHC
			Streptavidin TexasRed	Vector laboratories	SA-5006-1		5μg/mL	IHC-IF
Donkey	Anti-mouse		DyLight^{TM} 680	Invitrogen	SA5-10170		0.1μg/mL	WB
Goat	Anti-rabbit		DyLight^{TM} 800	Invitrogen	SA5-10036		0.1μg/mL	WB
Donkey	anti-goat		DyLight^{TM} 800	Invitrogen	SA5-10092		1μg/mL	WB

Supplementary Table 4. Real-time PCR primers

	Forward (5'-3')	Reverse (5'-3')
IL6	CCCACCGGGAACGAAAGA	TGGACCGAAGGCGCTTGT
CXCL8	AGTTTTTGAAGAGGGCTGAGA	TGCTTGAAGTTTCACTGGCATC
IL1B	TTCGAGGCACAAGGCACAA	TGGCTGCTTCAGACACTTGAG
ICAM1	AGCTTCGTGTCCTGTATGGC	TTTTCTGGCCACGTCCAGTT
SELE	TTCCAGCCTGCAATGTGGTT	GCTCTGGGCTCCCATTAGTT
ANGPT2	TCAGTGGCTAATGAAGCTTGAGA	CCGCTGTTTGGTTCAACAGG
ANGPT1	CGGCTACCATGCTGGAGATA	TCGAGAAGTTTGATTTAGTACCTGG
FLT1	CTGAACGTGGTTAACCTGCTG	TGTAGTGCTGCATCCTTGTTGA
F3	TTCCTAAGCCTCCGGGATGT	TGTTTGTTTTGGCTGTTTTCTTTCC
TFPI	ACAAAATAACCTCAACTCCGTTTT	GGCAACTCCGTATCTGTGAT
PLAT	TATTGCTGGTGCAACAGTGG	ACGTGGCCCTGGTATCTATT
PLAUR	GCCCTCGCGACATGGGT	CCTTCTTCCCACAAGCGCAC
SERPINE2	GAGCGCGGTCGTCCT	CCCGTGTTGGAGCCTAGTTC
SERPINB2	ACCAGAGAACAACCAGCATTTC	TCGGTGGTGCATAGCTTCACT
THBD	ACATCCTGGACGACGGTTTC	CGCAGATGCACTCGAAGGTA

Supplementary Table 5. Flow cytometry antibodies

Host Species	Species Reactivity	Antigen	Conjugate	Company	Catalog number	Clone	Concentration
Rat	Mouse	TER-119	N/A	BioLegend	116202	TER-119	1.25µg/mL
Rat	Human, Mouse	CD11b	N/A	BioLegend	101202	M1/70	0.63µg/mL
Rat	Mouse	Gr1 (Ly6G/Ly6C)	N/A	BioLegend	108402	RB6-8C5	0.63µg/mL
Rat	Mouse	B220 (CD45R)	N/A	BioLegend	103202	RA3-6B2	1.25µg/mL
Rat	Mouse	CD5	N/A	BioLegend	100602	53-7.3	0.63µg/mL
Rat	Mouse	CD3	N/A	BioLegend	100202	17A2	2.5µg/mL
Rat	Mouse	CD4	N/A	BioLegend	100402	RM4-4	0.63µg/mL
Rat	Mouse	CD8	N/A	BioLegend	100702	53-6.7	0.63µg/mL
Goat	Rat		PE-Cyanine5	eBioscience	A-10691	F(ab')2-IgG	2.5µg/mL
Rat	Mouse	cKit (CD117)	APC-eFluor780	eBioscience	47-1171-82	2B8	0.5µg/mL
Rat	Mouse	Sca-1	Pacific Blue	BioLegend	108120	D7	1.25µg/mL
Armenian hamster	Mouse	CD48	BV711	BioLegend	103439	HM48-1	2µg/mL
Rat	Mouse	CD150	PE	BioLegend	115904	TC15-12F12.2	0.5µg/mL
Rat	Mouse	Flk2	Biotin	BioLegend	13-1351-85	A2F10	5µg/mL
Rat	Mouse	CD34	FITC	eBioscience	11-0341-85	RAM34	20µg/mL
Rat	Mouse	FcγR	PerCP-eFluor710	eBioscience	46-0161-82	93	0.5µg/mL
Rat	Mouse	CD41	BV605	BioLegend	133921	MWReg30	0.5µg/mL
Rat	Mouse	CD105	APC	BioLegend	120413	MJ7/18	0.5µg/mL
Rat	Mouse	CD127	BV785	BioLegend	135037	A7R34	2µg/mL
			PE-Cyanine7 Streptavidin	BioLegend	405206	N/A	0.5µg/mL

Supplementary Figure 1

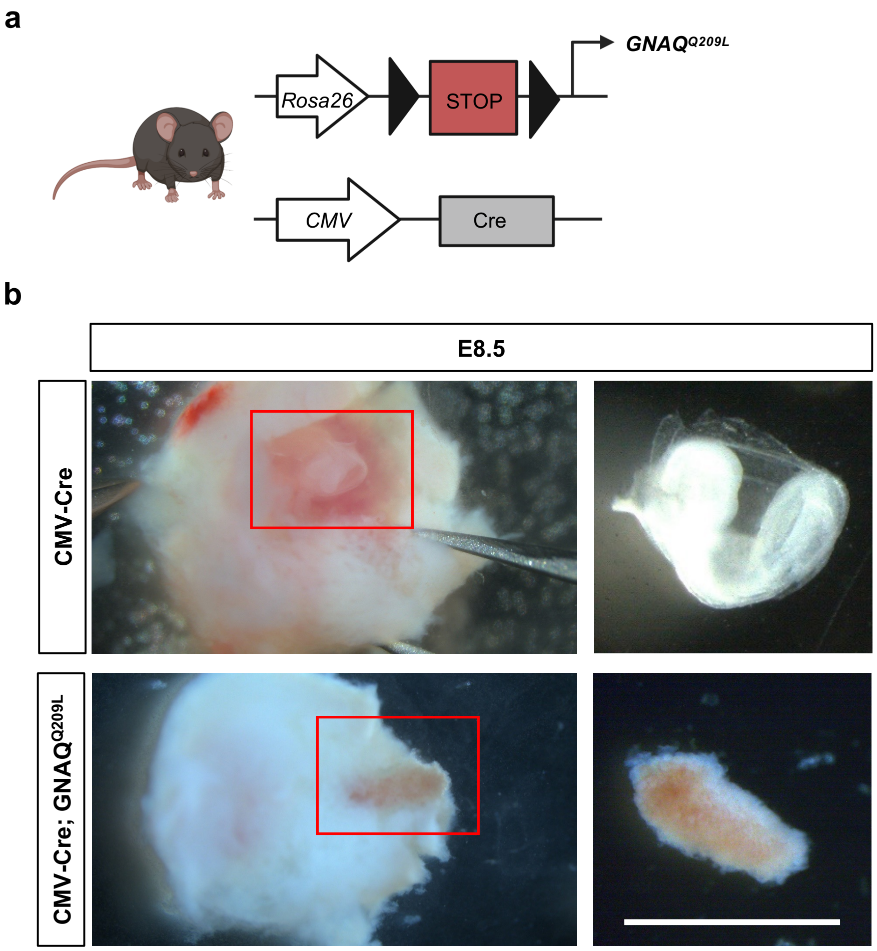

Supplementary Figure 1. Constitutive ubiquitous expression of *GNAQ*^{Q209L} results in embryonic lethality. (a) Breeding scheme. *CMV-Cre* mice were crossed with *Rosa26-floxed stop-GNAQ*^{Q209L}**.** Created with Biorender.com. **(b)** Representative images of *CMV-Cre*; *GNAQ*^{Q209L} and control *CMV-Cre* embryos dissected at E8.5. Mendelian ratios are shown in Supplementary Table 1. Scale bar: 1mm.

Supplementary Figure 2

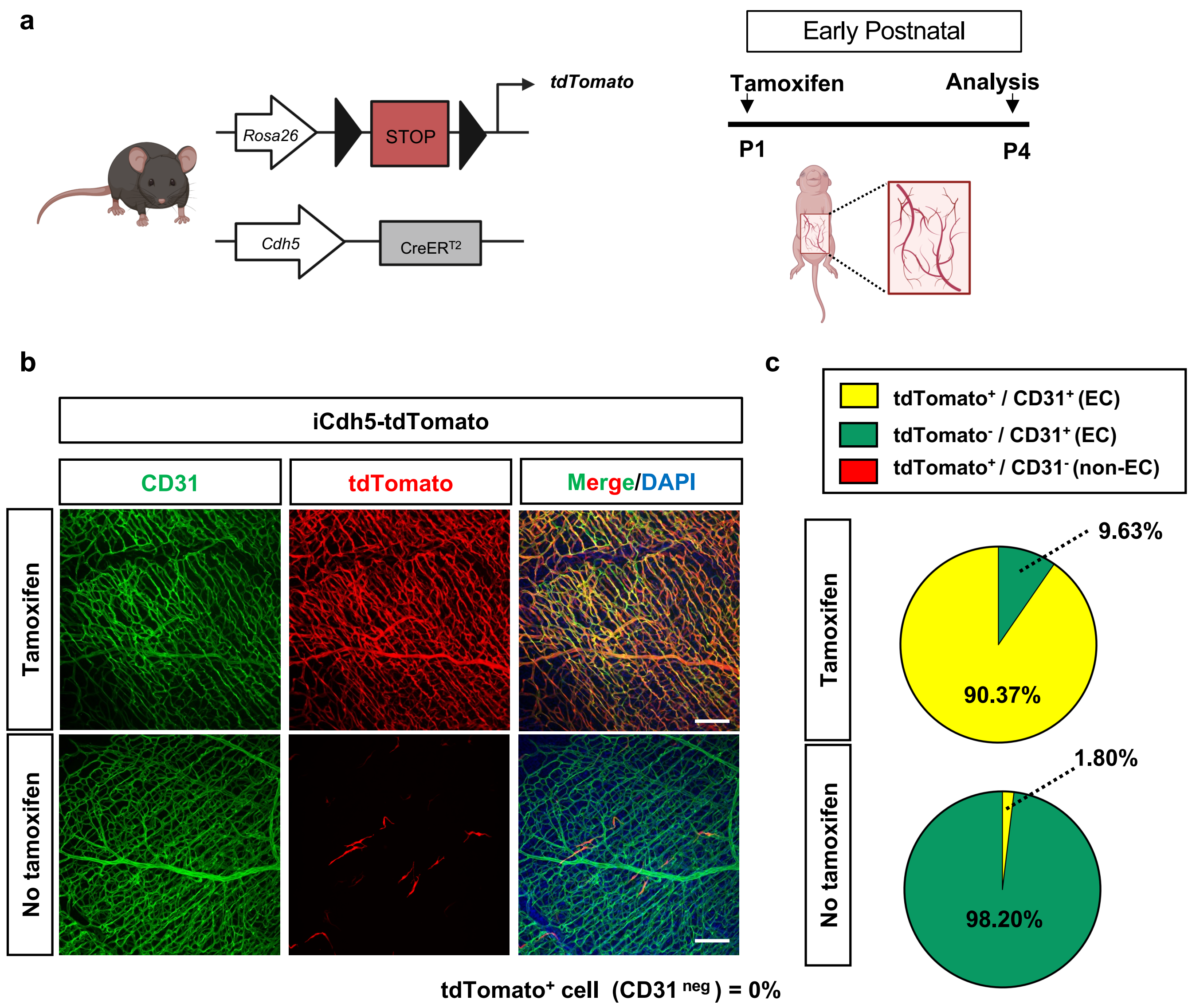

Supplementary Figure 2. Specificity of *Cdh5-CreER*^{T2}-mediated recombination in postnatal mice. **(a)** Breeding scheme. The *Rosa26-floxed stop-tdTomato* reporter mouse was crossed with *Cdh5-iCreER*^{T2} to generate a mouse with EC specific tdTomato expression (*iCdh5-tdTomato*). Schematic describing tamoxifen administration at postnatal (P1) and experimental analysis of vasculature in the subcutaneous tissue at P4. Created with BioRender.com. **(b)** Representative z-stack confocal images (max intensity projections) showing CD31 (green) and tdTomato (red) expression in subcutaneous whole-mounts of tamoxifen-treated (upper panel) and untreated (lower panel) *iCdh5-tdTomato* mice. DAPI in blue for nuclear staining. Scale bar: 100µm. **(c)** Number of CD31^{+} (green), tdTomato^{+} (red) and double positive cells (yellow) was analyzed. Quantification of percentage of tdTomato^{+} CD31^{+} EC (yellow) and tdTomato ^{negative} CD31^{+} EC (green) shown in a pie chart (mean of n=3 tamoxifen treated mice and n=4 no tamoxifen treated mice). tdTomato^{+}/CD31^{negative} cells were not detected. Source data are provided in Source Data file.

Supplementary Figure 3

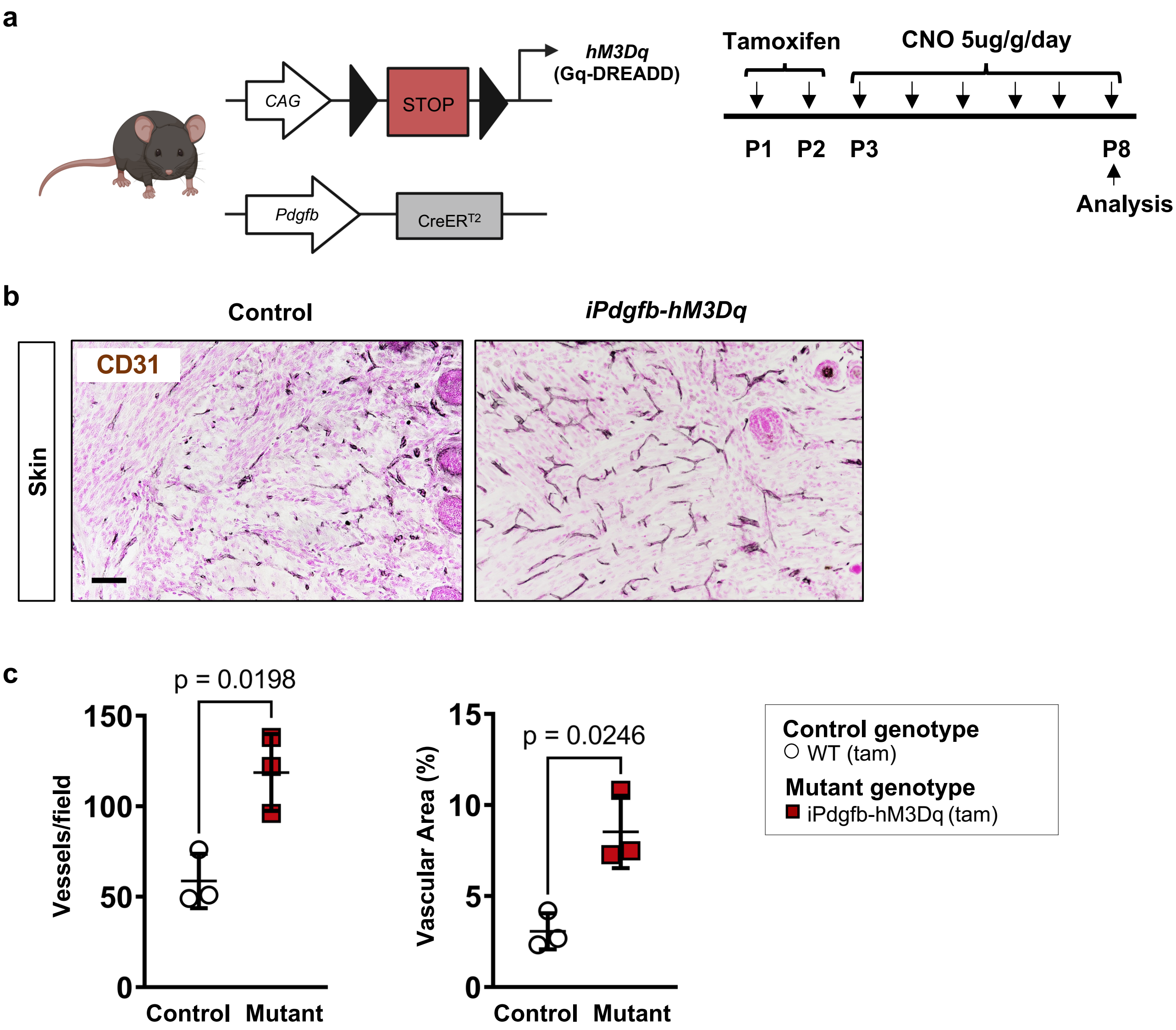

Supplementary Figure 3. Endothelial-specific hyperactive Gαq signaling in Gαq-DREADD mice results in increased vascularity. (a) Breeding scheme for Gαq-DREADD mice with tamoxifen, and clozapine N-oxide (CNO) injection diagram. Created with Biorender.com. **(b)** Representative images of CD31 (dark brown) immunohistochemical staining in murine skin from Gαq hyperactive *iPdgfb-hM3Dq* (*CAG-LSL-Gαq-DREADD; Pdgfb-iCreER^{T2}*) mice and control littermates. Scale bar: 50μm **(c)** Quantifications of skin vascular density (vessels/field) and vascular area as percentage of total field area; n=3 mice/group, mean±SD, unpaired two-tailed Welch's t-test. Source data are provided in Source Data file.

Supplementary Figure 4

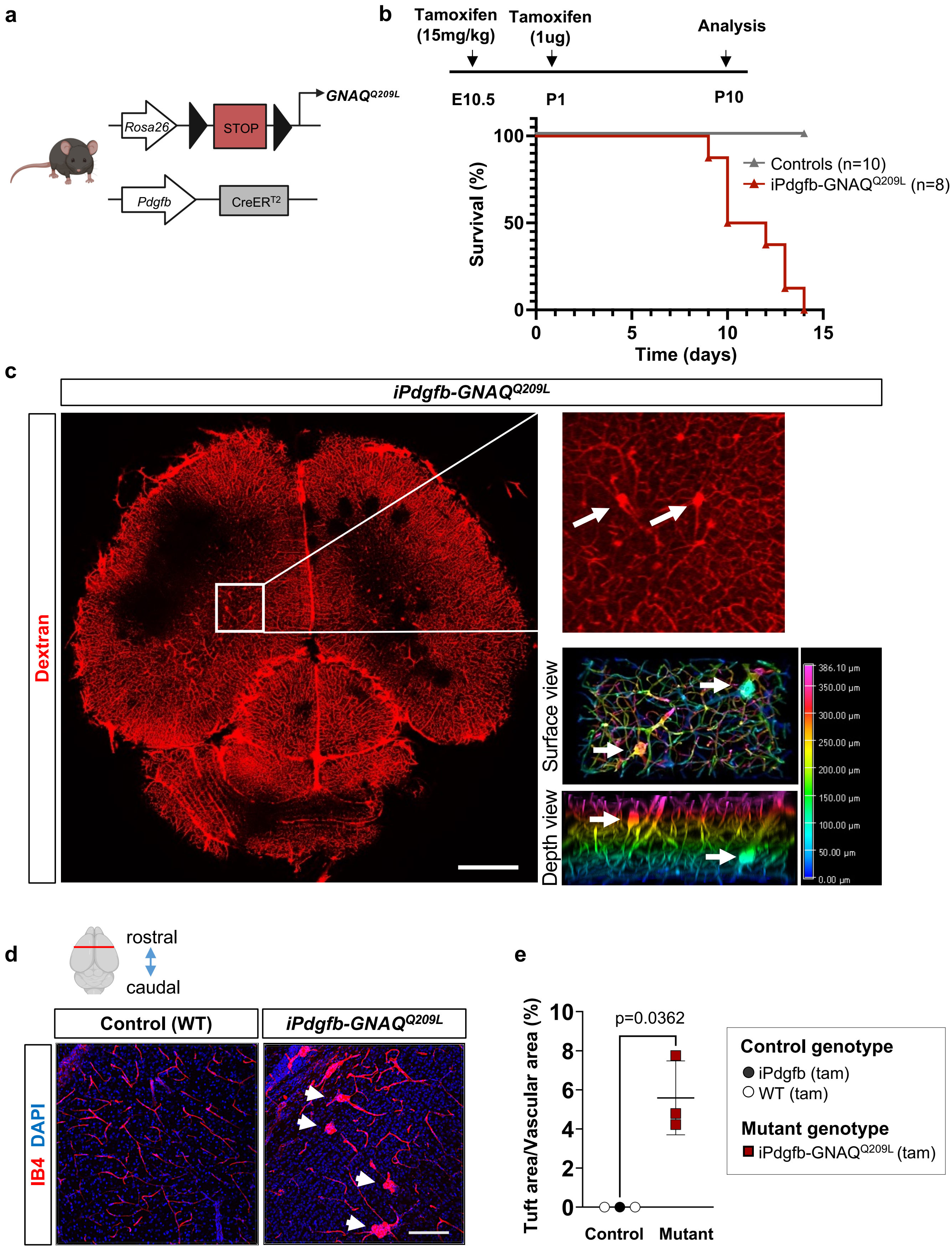

Supplementary Figure 4. Endothelial-specific *GNAQ*^{Q209L} expression results in the formation of vascular tufts in the brain. (a) Breeding scheme. The *Rosa26-floxed stop-GNAQ*^{Q209L} mouse was crossed with *Pdgfb-iCreER*^{T2} to generate mice with EC specific *GNAQ*^{Q209L} expression. Created with Biorender.com. **(b)** Schematic of tamoxifen induction and time of brain dissection for analysis. Tamoxifen injections were given to pregnant dams at E10.5, and in pups at P1. Kaplan-Meier curve comparing the survival percentage of *iPdgfb-GNAQ*^{Q209L} (n=8, red) to control (n=10, gray) mice. Gehan–Breslow–Wilcoxon test ($p<0.0001$). **(c)** On the left: Whole brain of *iPdgfb-GNAQ*^{Q209L} P10 pup injected with Dextran 70kD and imaged with two-photon microscopy after passive clarity technique. Scale bar: 200μm. On the right, top: magnified image from inset; bottom: Imaris 3D reconstructions with depth coloring and relative scale. White arrows point to vascular tufts. **(d)** IB4 labeling (in red) in mouse brain section shows vascular abnormalities (tufts) in *iPdgfb-GNAQ*^{Q209L} P10 pups (DAPI in blue for nuclear staining). White arrows indicate vascular tufts. Scale bar: 100μm. Schematic created with Biorender.com. **(e)** Quantification of tuft area normalized to the vascular area; n=3 mice/group, mean±SD, unpaired two-tailed Welch's t-test. Mouse genotypes are indicated. Source data for (b,e) are provided in Source Data file.

Supplementary Figure 5

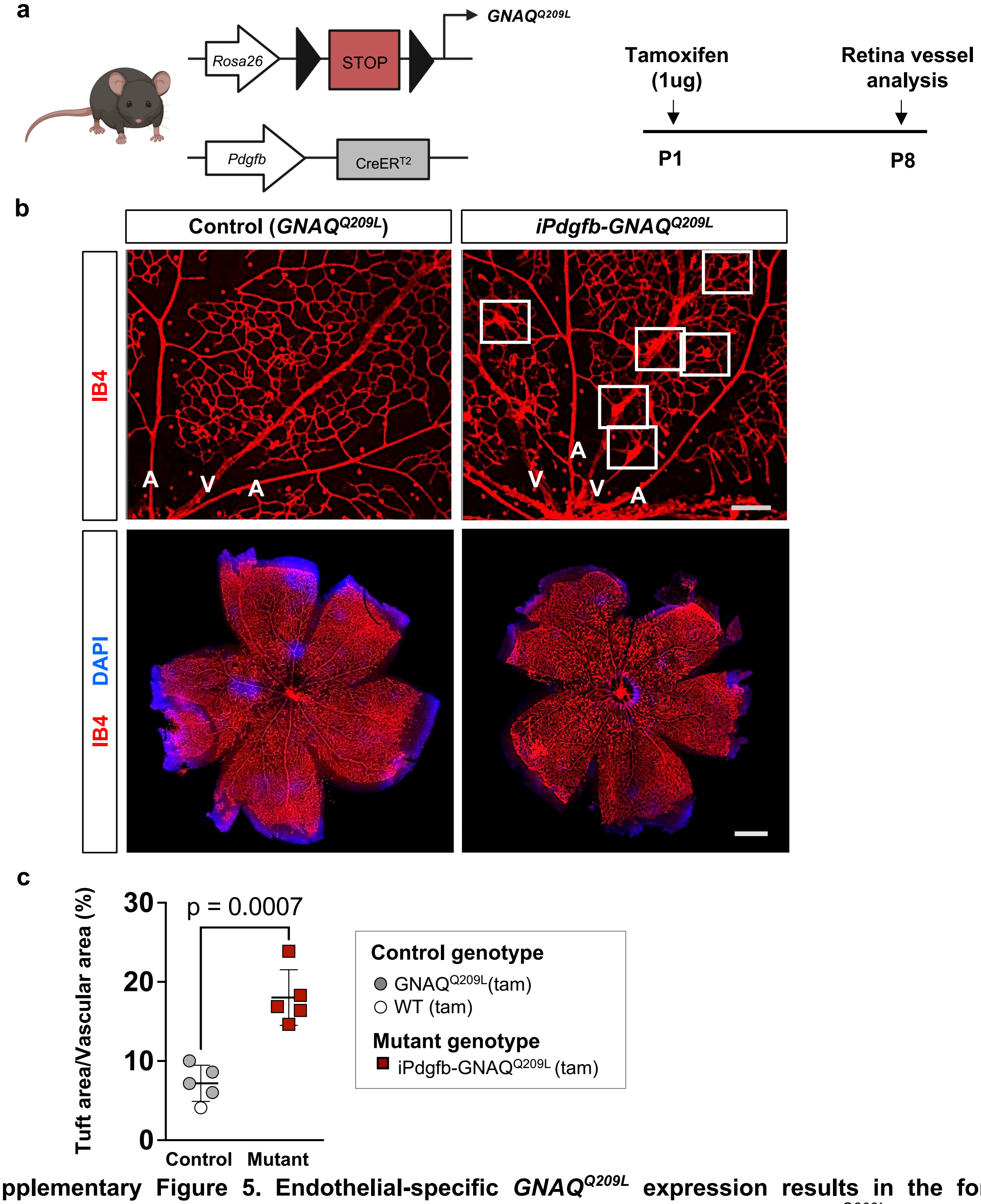

Supplementary Figure 5. Endothelial-specific *GNAQ^{Q209L}* expression results in the formation of vascular tufts in the retina. (a) Breeding scheme. The *Rosa26-floxed stop-GNAQ^{Q209L}* mouse was crossed with *Pdgfb-iCreER^{T2}* to generate EC specific *GNAQ^{Q209L}* expression. Created with Biorender.com. Schematic of tamoxifen induction and time of retina dissection for analysis. Tamoxifen injections were given at P1 for the analysis of vascular development in *iPdgfb-GNAQ^{Q209L}* mouse retinas. Retinas were dissected and analyzed at day 8 (P8). **(b)** Flat-mounted IB4 (Isolectin B4)-stained retinas (red). White boxes bring attention to vascular tufts in the top panel. Vascular tufts can be detected in capillaries and veins (V) but not arteries (A). DAPI labels nuclei in bottom panels. Scale bars: 200μm (top) and 500μm (bottom). **(c)** Vascular tuft area was quantified as the area occupied by IB4^{+} tufts/total vascular area; *iPdgfb-GNAQ^{Q209L}* mice and control littermates (n= 5 mice/group, 2 eyes analyzed for each mouse and averaged), mean±SD, unpaired two-tailed Welch's t-test. Mouse genotypes are indicated. Source data are provided in Source Data file.

Supplementary Figure 6

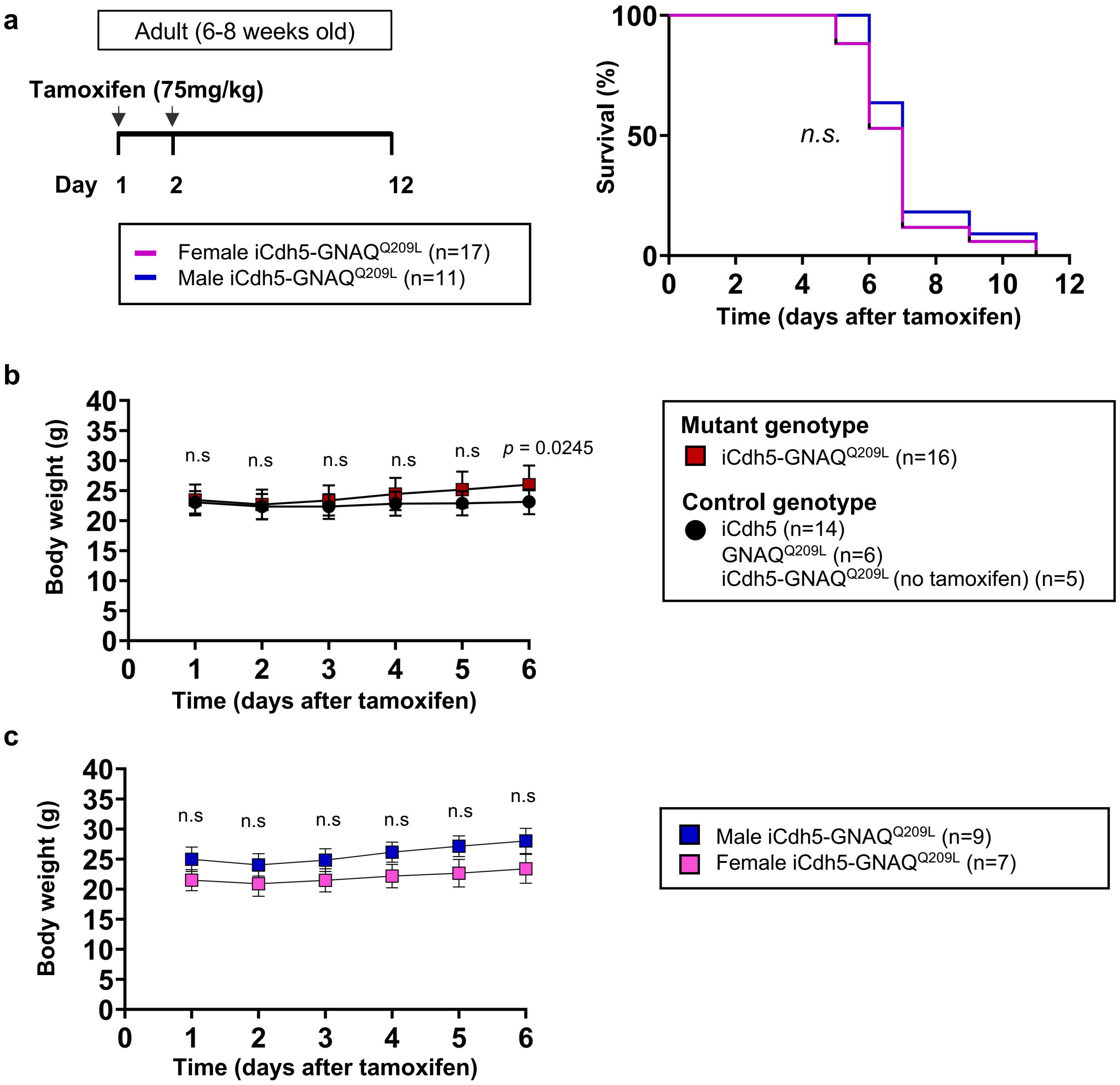

Supplementary Figure 6. No differences in male vs female survival of adult *iCdh5-GNAQ^{Q209L}* mice. (a) Tamoxifen induction scheme. Kaplan-Meier curve comparing the survival percentage of *iCdh5-GNAQ^{Q209L}* female (n=17, pink) to male (n=11, blue) mice. Gehan–Breslow–Wilcoxon test. **(b)** Graph showing bodyweight of *iCdh5-GNAQ^{Q209L}* (n=16) and control littermates (n=25) over 6 days after first tamoxifen injection. Mouse genotypes are indicated; mean±SD, two-way ANOVA (Šídák's multiple comparisons test). **(c)** Graph comparing the bodyweight of *iCdh5-GNAQ^{Q209L}* female (n=7, pink) to male (n=9, blue) mice over 6 days after first tamoxifen injection, mean±SD, two-way ANOVA (Šídák's multiple comparisons test). n.s. for p<0.05. Source data for (a-c) are provided in Source Data file.

Supplementary Figure 7

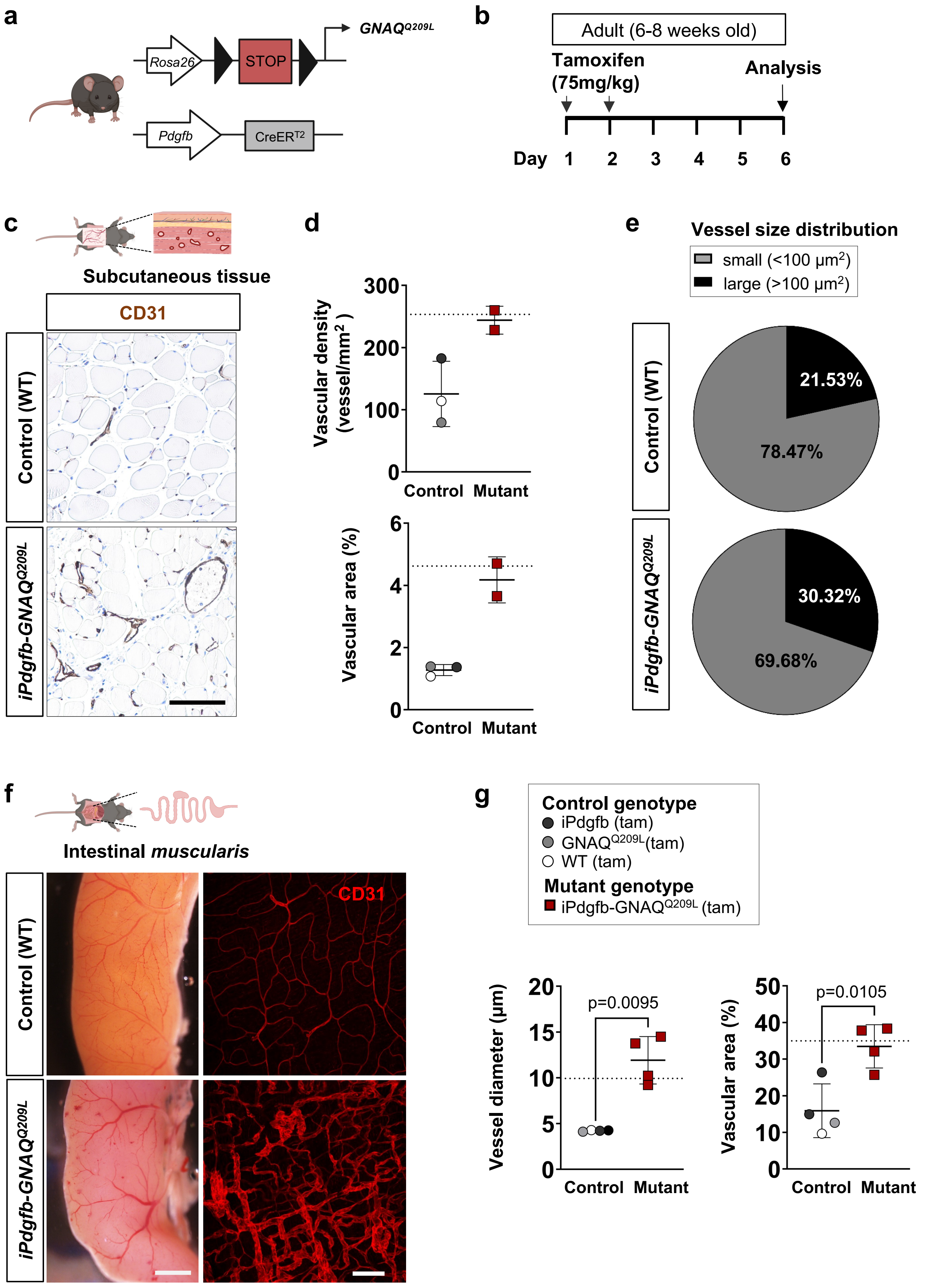

Supplementary Figure 7. *iPdgfb-GNAQ^{Q209L}* mice show phenotypes similar to *iCdh5-GNAQ^{Q209L}* in the subcutaneous and intestinal vasculature. **(a)** Breeding scheme. The *Rosa26-floxed stop-GNAQ^{Q209L}* mouse was crossed with *Pdgfb-iCreER^{T2}* to generate EC-specific *GNAQ^{Q209L}* expression. **(b)** Schematic describing tamoxifen regimen for adult mice (6-8 weeks of age). **(c)** Representative images of subcutaneous tissue sections from tamoxifen-treated *iPdgfb-GNAQ^{Q209L}* and WT littermate control mouse immuno-stained for CD31 (dark brown), scale bar: 50μm. **(d)** Quantification of vascular density defined as number of vessels per area and vascular area as percentage of total area; *iPdgfb-GNAQ^{Q209L}* (n=2) and control animals (n=3), mean±SD. **(e)** Vessel size distribution as percentage of small (<100 μm^2) and large (>100 μm^2) vessels. *iPdgfb-GNAQ^{Q209L}* (n=2) and control WT animals (n=3). **(f)** Representative macroscopic photographs of intestine of tamoxifen-treated *iPdgfb-GNAQ^{Q209L}* and WT control mice. Scale bar: 1mm. Representative z-stack confocal images (max. intensity projections) of intestinal *muscularis* stained for CD31 (red). Scale bar: 100μm. **(g)** Quantification of vessel diameter and vascular area as percentage of tissue area. *iPdgfb-GNAQ^{Q209L}* (n=4) and control littermates (n=4), mean±SD, unpaired two-tailed Welch's t-test. Mouse genotypes are indicated. Dashed lines indicated mean values for *iCdh5-GNAQ^{Q209L}* as from Fig.2. Schematics in (a,c,f) were created with BioRender.com. Source data for (d,e,g) are provided in Source Data file.

Supplementary Figure 8

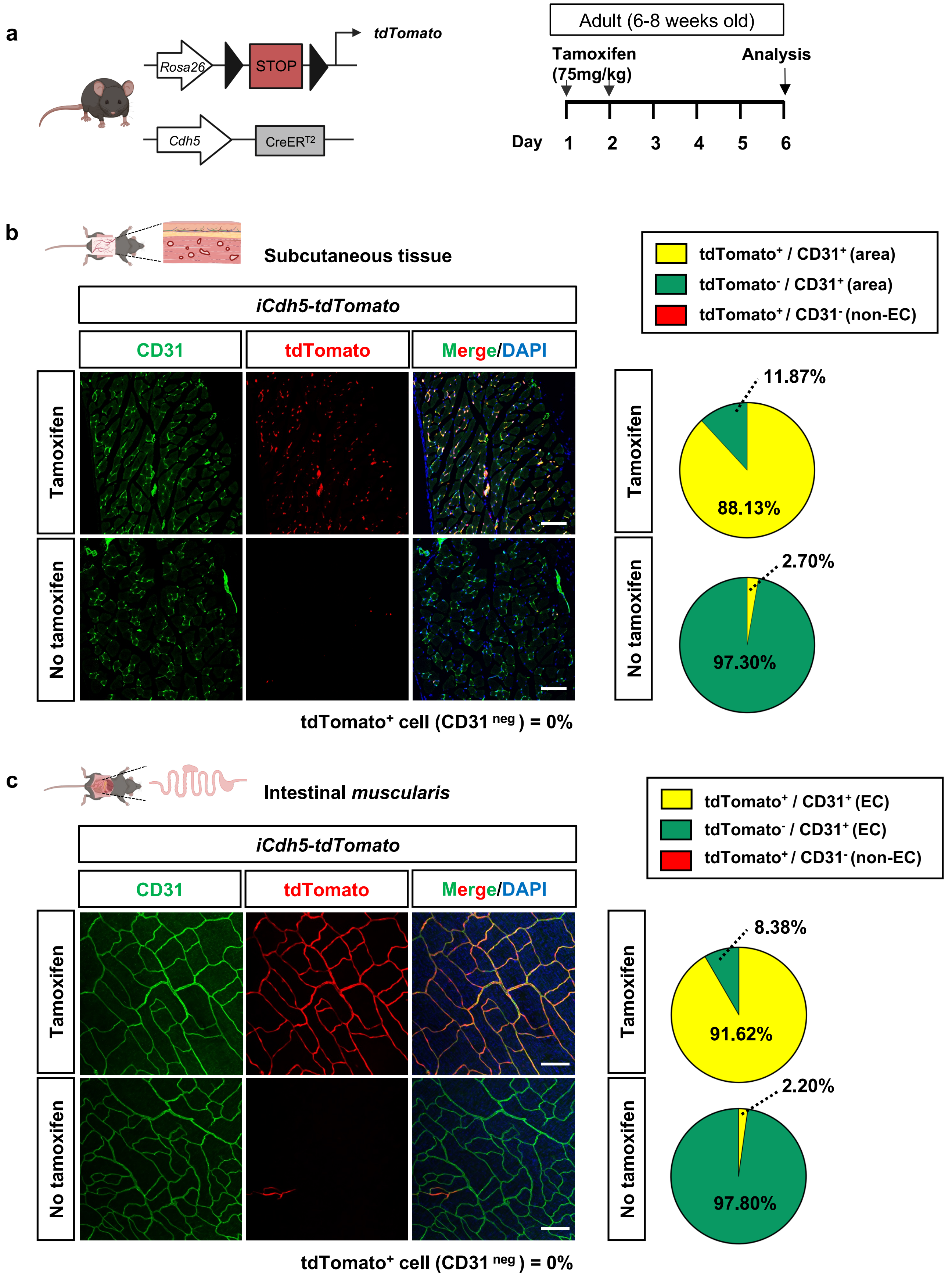

Supplementary Figure 8. Specificity of *iCdh5-CreER^{T2}*-mediated recombination in adult mice (a) Breeding scheme. The *Rosa26-floxed stop-tdTomato* reporter mouse was crossed with *Cdh5-iCreER^{T2}* to generate a mouse with EC specific tdTomato expression (*iCdh5-tdTomato*). Schematic describing tamoxifen regimen for adult mice (6-8 weeks) before experimental analysis of vasculature in the subcutaneous tissue and intestinal tissue. **(b)** tdTomato expression (red) and CD31 staining (green) in subcutaneous tissue sections of tamoxifen treated (upper panel) and untreated (lower panel) *iCdh5-tdTomato* mice. Z-stack confocal images (max. intensity projections). Scale bar: 100μm. Quantification of percentage of tdTomato^{+} (yellow) and tdTomato^{-} (green) vessel area is shown in a pie chart (n=2 mice per group). **(c)** tdTomato expression (red) and CD31 staining (green) in intestinal *muscularis* of tamoxifen treated (upper panel) and untreated (lower panel). Whole-mount confocal z-stack images (max. intensity projections). Quantification of percentage of tdTomato^{+} (yellow) EC and tdTomato^{negative} (green) EC shown in a pie chart (n=2 mice per group). No tdTomato^{+}/CD31^{negative} cells (non-EC, red) were detected. Scale bar: 100μm. In (b,c): DAPI in blue for nuclear staining. Schematics in (a,b,c) were created with BioRender.com. Source data for (b-c) are provided in Source Data file.

Supplementary Figure 9

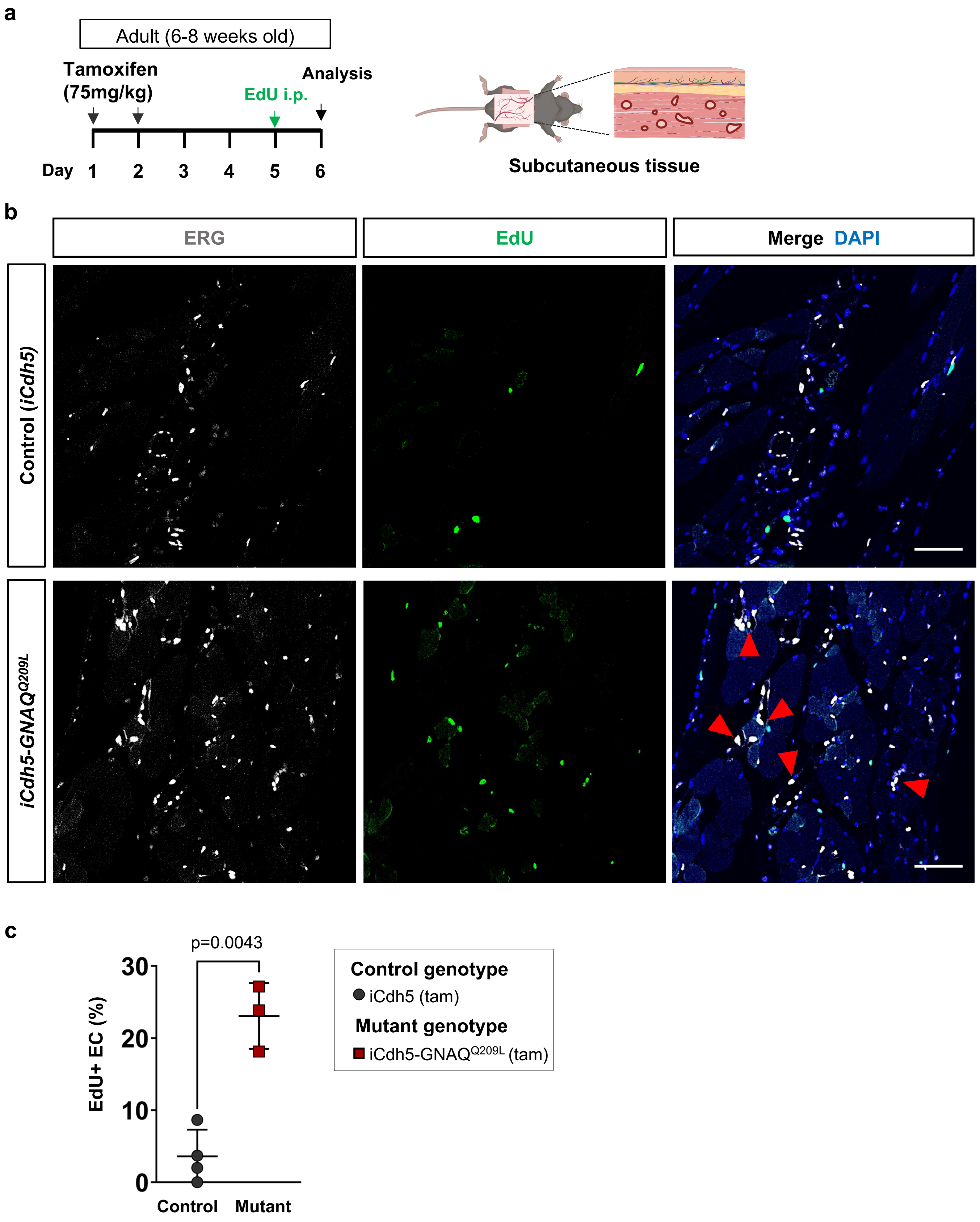

Supplementary Figure 9. ***GNAQ*** ^{Q209L} **expression results in increased EC proliferation in the subcutaneous tissue. (a)** Tamoxifen induction and EdU injection scheme. EdU was administered to *iCdh5-GNAQ* ^{Q209L} (n=3) and *iCdh5* control littermates (n=4), intraperitoneally 24h before sacrifice. Schematic was created with BioRender.com. **(b)** Subcutaneous tissue sections were labeled for EdU (green) along with ERG antibody to label EC nuclei (white). Red arrowheads indicate ERG^+ EdU^+ events. DAPI in blue for nuclear staining. Scale bar: 50μm. **(c)** The number of EdU positive endothelial cells (EdU^+/ERG^+) was counted in *iCdh5-GNAQ* ^{Q209L} (n=3) and control littermates (n=4) and is shown as percentage of total number of ECs (ERG^+), mean±SD, unpaired two-tailed Welch's t-test. Mouse genotypes are indicated. Source data for (c) are provided as Source Data file.

Supplementary Figure 10

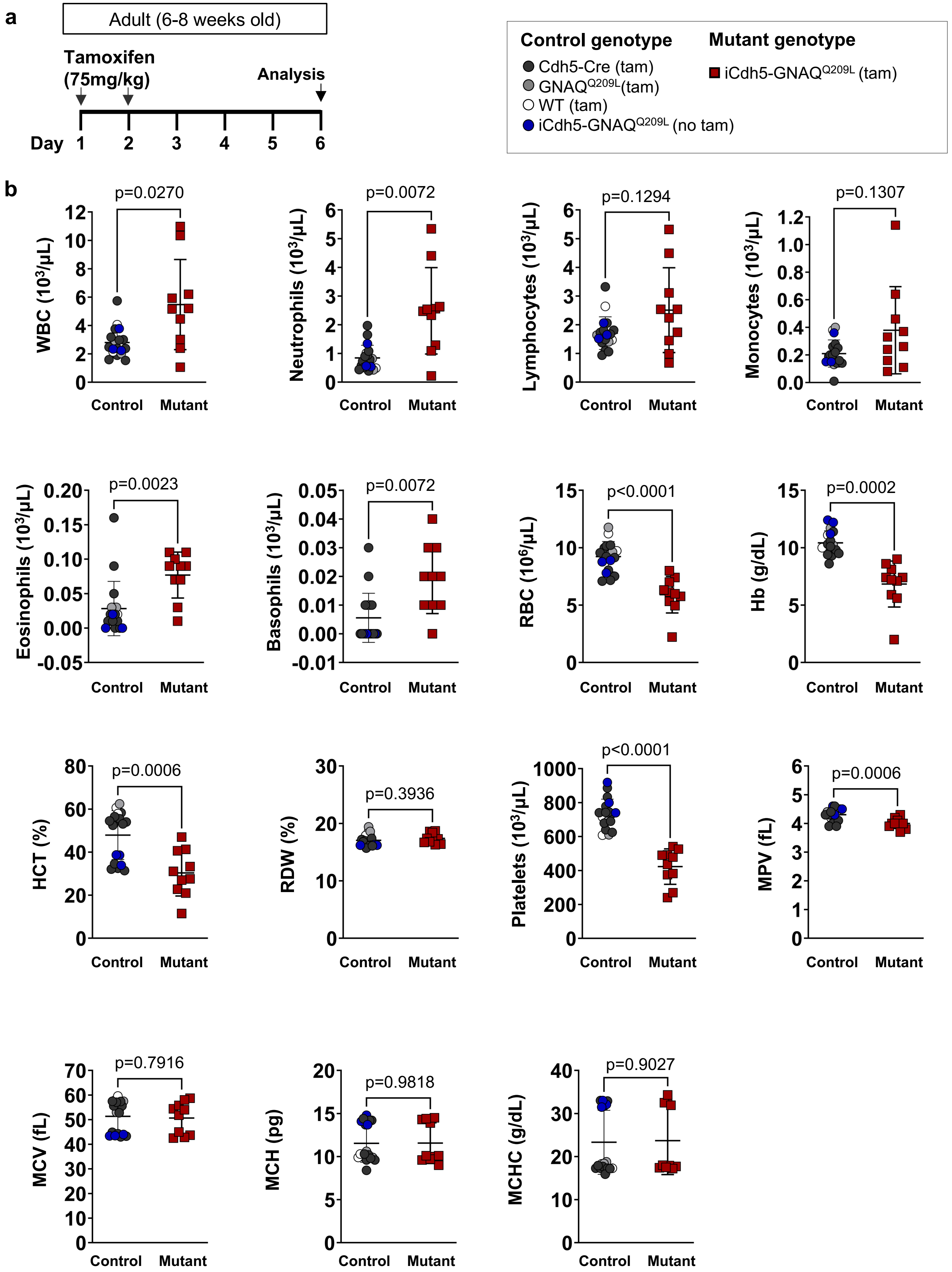

Supplementary Figure 10. Complete Blood Counts (CBC) revealed thrombocytopenia and anemia in *iCdh5-GNAQ*^{Q209L} mice. (a) Tamoxifen induction scheme. **(b)** Hematological parameters of *iCdh5-GNAQ*^{Q209L} (n=10) and control (n=18) mice, mean±SD, unpaired two-tailed Welch's t-test. Mouse genotypes are indicated. WBC, white blood cells; RBC, red blood cells; Hb, hemoglobin; HCT, hematocrit; MCV, mean corpuscular volume; MCH, mean cellular hemoglobin; MCHC, mean corpuscular/cellular hemoglobin concentration; RDW-CV, red blood cell distribution width - coefficient of variation; MPV, mean platelet volume. Source data are provided as Source Data file.

Supplementary Figure 11

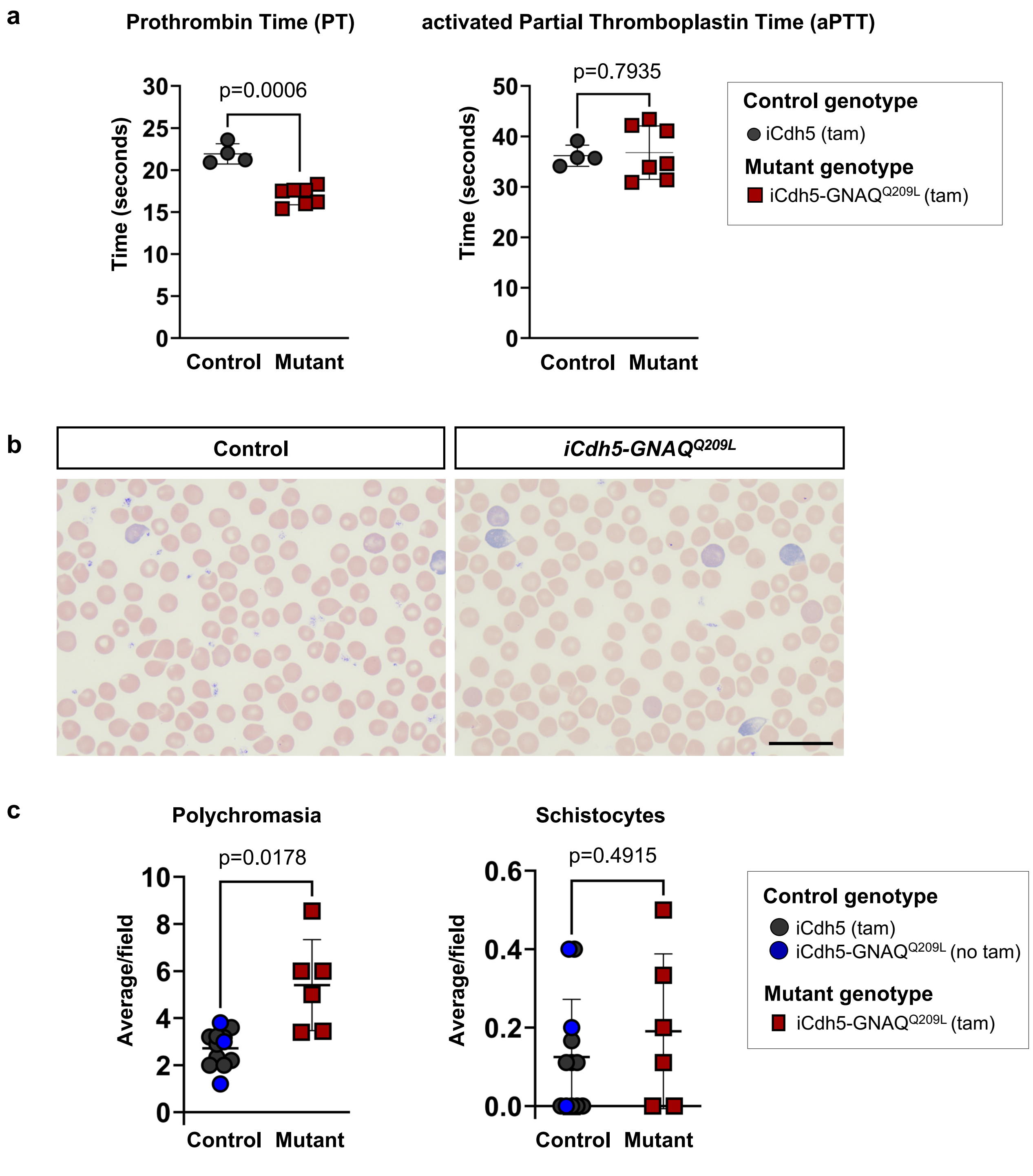

Supplementary Figure 11. Hemostasis analysis revealed reduced prothrombin time and increased polychromasia. (a) Prothrombin time (PT) and activated partial thromboplastin time (aPTT) in *iCdh5-GNAQ*^{Q209L} mice (n=7) compared to control (n=4) littermates. Mean±SD, unpaired two-tailed Welch`s t-test. **(b)** Representative blood smear in *iCdh5-GNAQ*^{Q209L} and control WT mouse. Scale bar: 20µm. **(c)** Quantification of schistocytes number and polychromasia events/field; n=12 controls, n=6 mutants. Mouse genotypes are indicated. Mean±SD, unpaired two-tailed Welch's t-test. Source data for (a,c) are provided in Source Data file.

Supplementary Figure 12

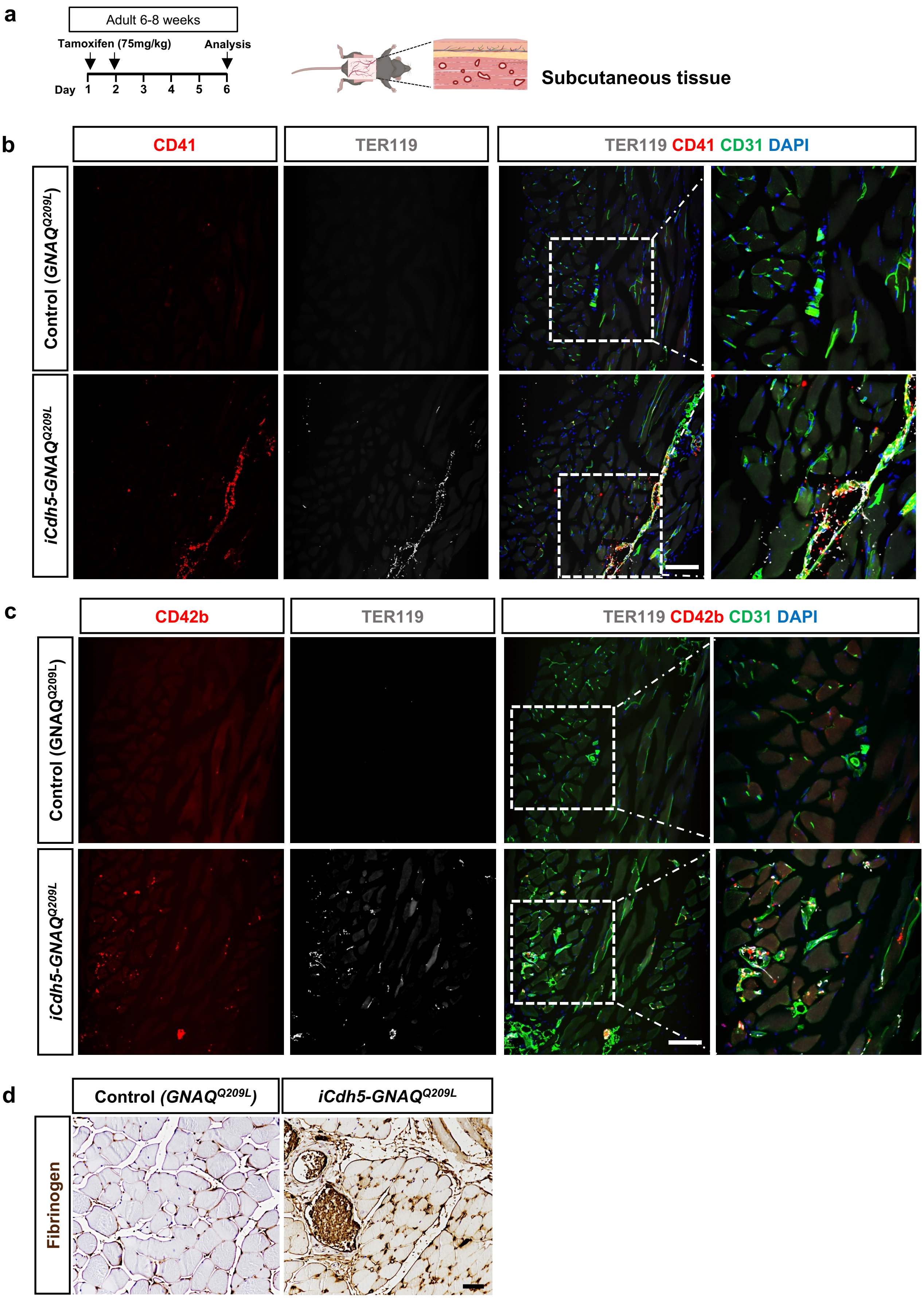

Supplementary Figure 12. Platelet and Fibrinogen accumulation in vascular tufts of *iCdh5-QNAQ*^{Q209L} mice. (**a**) Schematic of tamoxifen induction scheme. Schematic was created with BioRender.com. **(b, c)** Representative confocal z-stack images (max. intensity projection) of subcutaneous tissue (n=3 mice/group) stained for CD41 (top) or CD42b (bottom) (red) together with CD31 (green) and Ter119 (white). DAPI in blue for nuclear staining. Scale bar: 100µm. **(d)** Representative immunohistochemistry staining for fibrinogen (dark brown) (n=8 *iCdh5-GNAQ*^{Q209L} mice, n=7 control litternates). Scale bar: 50µm.

Supplementary Figure 13

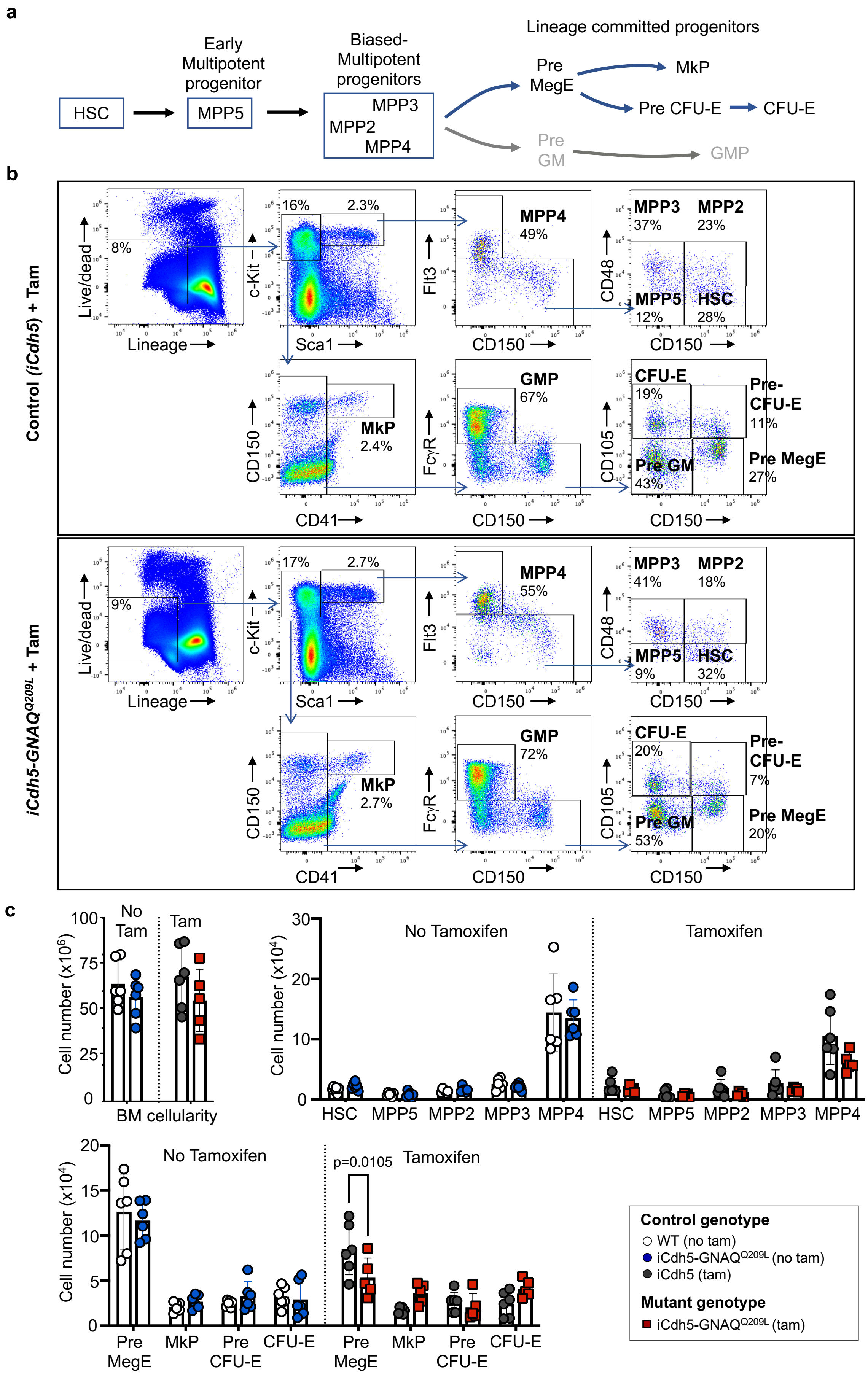

Supplementary Figure 13. Bone marrow (BM) hematopoietic stem cell analysis in *iCdh5-GNAQ*^{Q209L} mice. (**a**) Schematic describing the BM hematopoietic hierarchy including (i) the hematopoietic stem cells (HSC), (ii) the early multipotent progenitors (MMP2-5), and (iii) lineage-committed progenitors. This latter group includes stages of granulocyte/macrophage differentiation denoted Pre GM and GMP and a hierarchy defining the megakaryocyte/erythroid differentiation (common MegE progenitors [Pre MegE], erythroid colony-forming unit [Pre CFU-E and CFU-E] and megakaryocyte [MkP] precursors). (**b**) Representative flow cytometry plots describing the hematopoietic hierarchy present in *iCdh5* and *iCdh5-GNAQ*^{Q209L} mice, 6 days after tamoxifen treatment (Tam). (**c**) BM cellularity and cell number of the stem and progenitor compartments present in control (n=6 WT (no tam), and n=6 *iCdh5-GNAQ*^{Q209L} (no tam)) and tamoxifen-induced control (n=6 *iCdh5* (tam)) and mutant (n=5 *iCdh5-GNAQ*^{Q209L} (tam)) mice, 6 days after vehicle- or tamoxifen-treatment, mean±SD, two-way ANOVA with Sidak’s post hoc test for multiple comparisons. Source data for (c) are provided in Source Data file.

Supplementary Figure 14

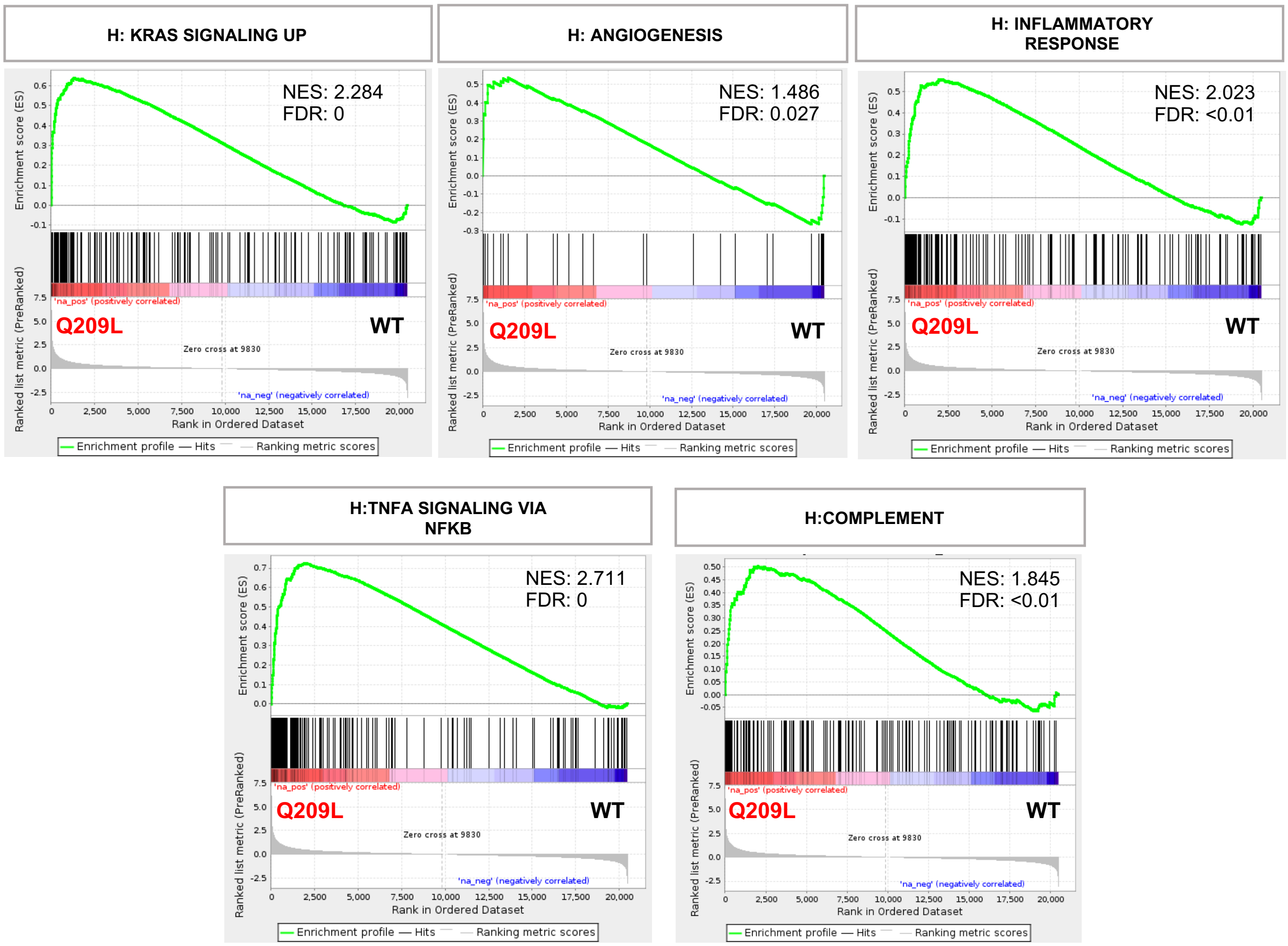

Supplementary Figure 14. Transcriptomic analysis in EC expressing *GNAQ*-Q209L or *GNAQ*-WT. Gene Set Enrichment Analysis (GSEA) was performed on normalized RNA-Sequencing expression data from n=4 independent samples of iEC *GNAQ*-Q209L (left, red) compared to n=4 iEC *GNAQ*-WT (right, blue) utilizing the Hallmark Database. Pathways related to KRAS Signaling Up, Angiogenesis, Inflammatory Response, TNFA Signaling via NFKB and Complement. Enrichment plots are displayed with the FDR (adjusted) p-value and normalized enrichment score (NES). Source data are provided in Source Data file.

Supplementary Figure 15

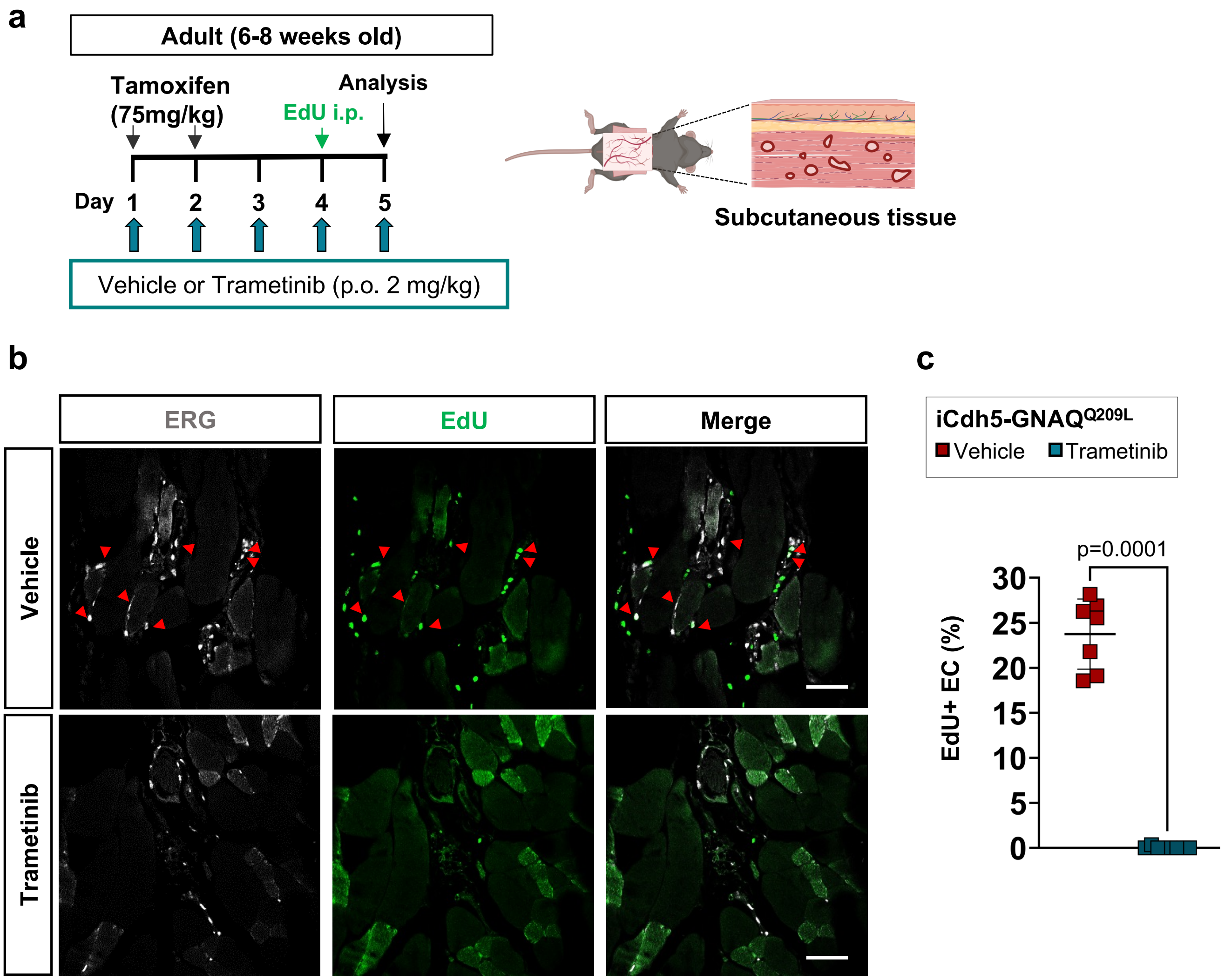

Supplementary Figure 15. Trametinib treatment reduces EC proliferation in the subcutaneous vasculature of *iCdh5-GNAQ*^{Q209L} mice. (a) Tamoxifen induction, Trametinib treatment (daily oral gavage, per os (p.o.)) and EdU injection scheme. EdU was administered to vehicle-treated *iCdh5-GNAQ*^{Q209L} (n=7) and Trametinib-treated *iCdh5-GNAQ*^{Q209L} (n=8), intraperitoneally 24h before analysis. Schematic was created with BioRender.com. **(b)** Subcutaneous tissue sections were labeled for EdU (green) along with ERG antibody staining (white) for EC nuclei. Red arrowheads indicate EdU positive endothelial cells. Scale bar: 50μm. **(c)** The number of EdU positive endothelial cells (EdU^+/ERG^+) were counted and shown as percentage of total number of endothelial cells (ERG^+), mean±SD, unpaired two-tailed Welch's t-test. Source data for (c) are provided in Source Data file.

Supplementary Figure 16

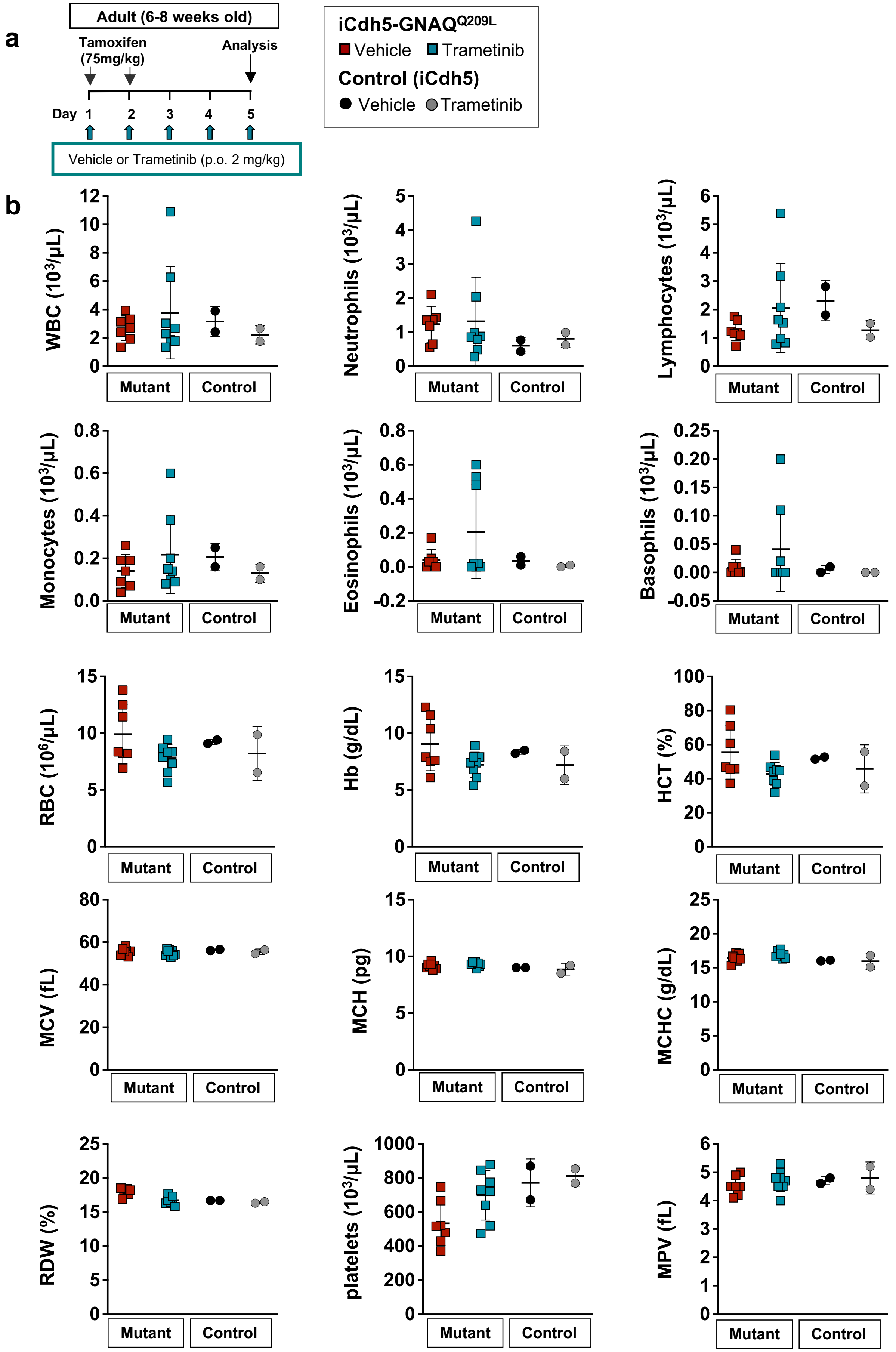

Supplementary Figure 16. Complete Blood Counts (CBC) in *iCdh5-GNAQ*^{Q209L} mice treated with Trametinib. (a) Tamoxifen induction and Trametinib treatment (daily oral gavage, per os (p.o.)) scheme. **(b)** Hematological parameters of *iCdh5-GNAQ*^{Q209L} and control mice treated with vehicle or trametinib. WBC, white blood cells; RBC, red blood cells; Hb, hemoglobin; HCT, hematocrit; MCV, mean corpuscular volume; MCH, mean cellular hemoglobin; MCHC, mean corpuscular/cellular hemoglobin concentration; RDW-CV, red blood cell distribution width - coefficient of variation; MPV, mean platelet volume. *iCdh5-GNAQ*^{Q209L} Vehicle (n=7) and Trametinib (n=8) treated mice, mean$\pm$SD. Control mice (*iCdh5*) were also treated with Vehicle (n=2) or Trametinib (n=2). Source data for (b) are provided in Source Data file.

Supplementary Figure 17

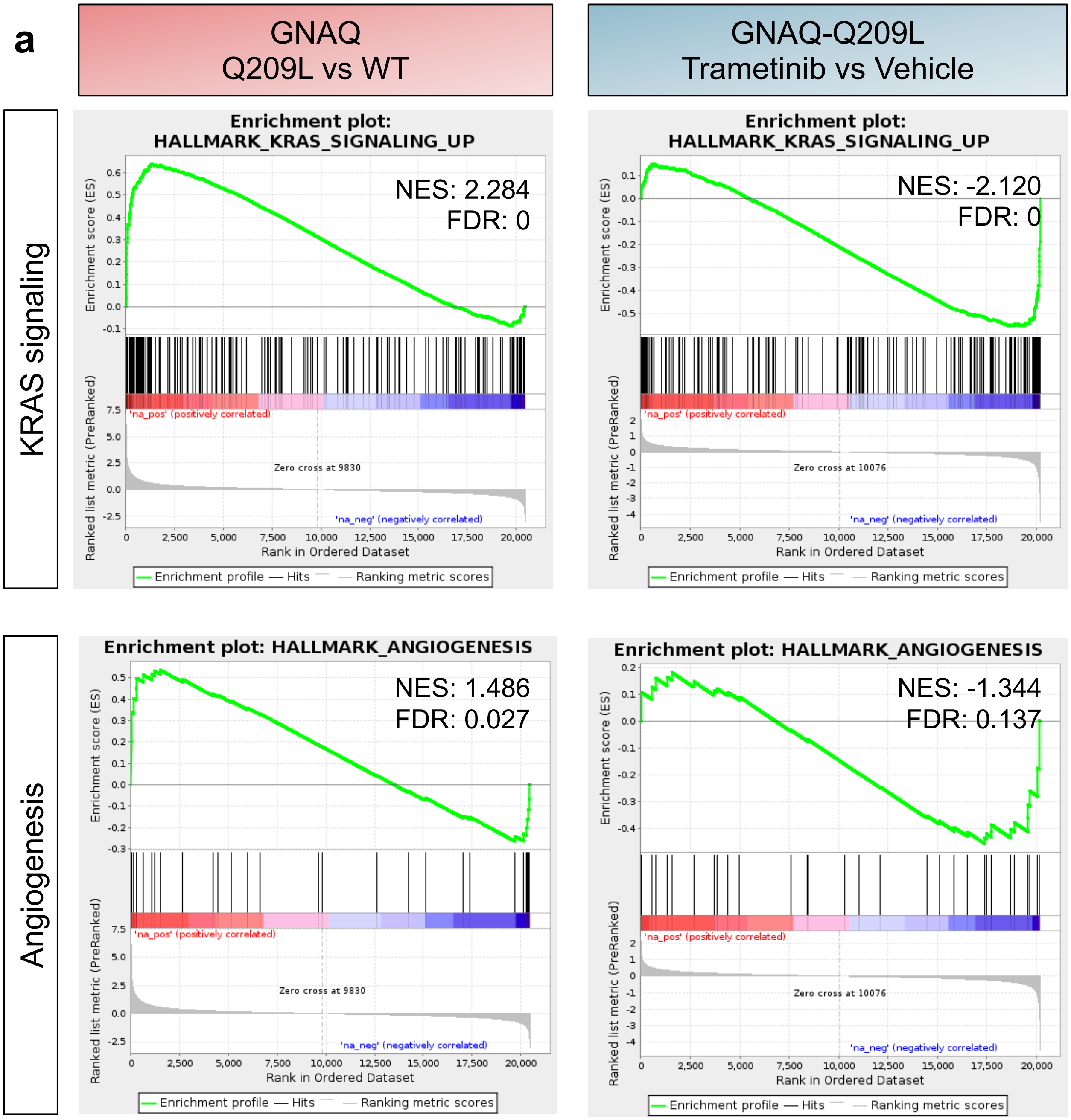

Supplementary Figure 17

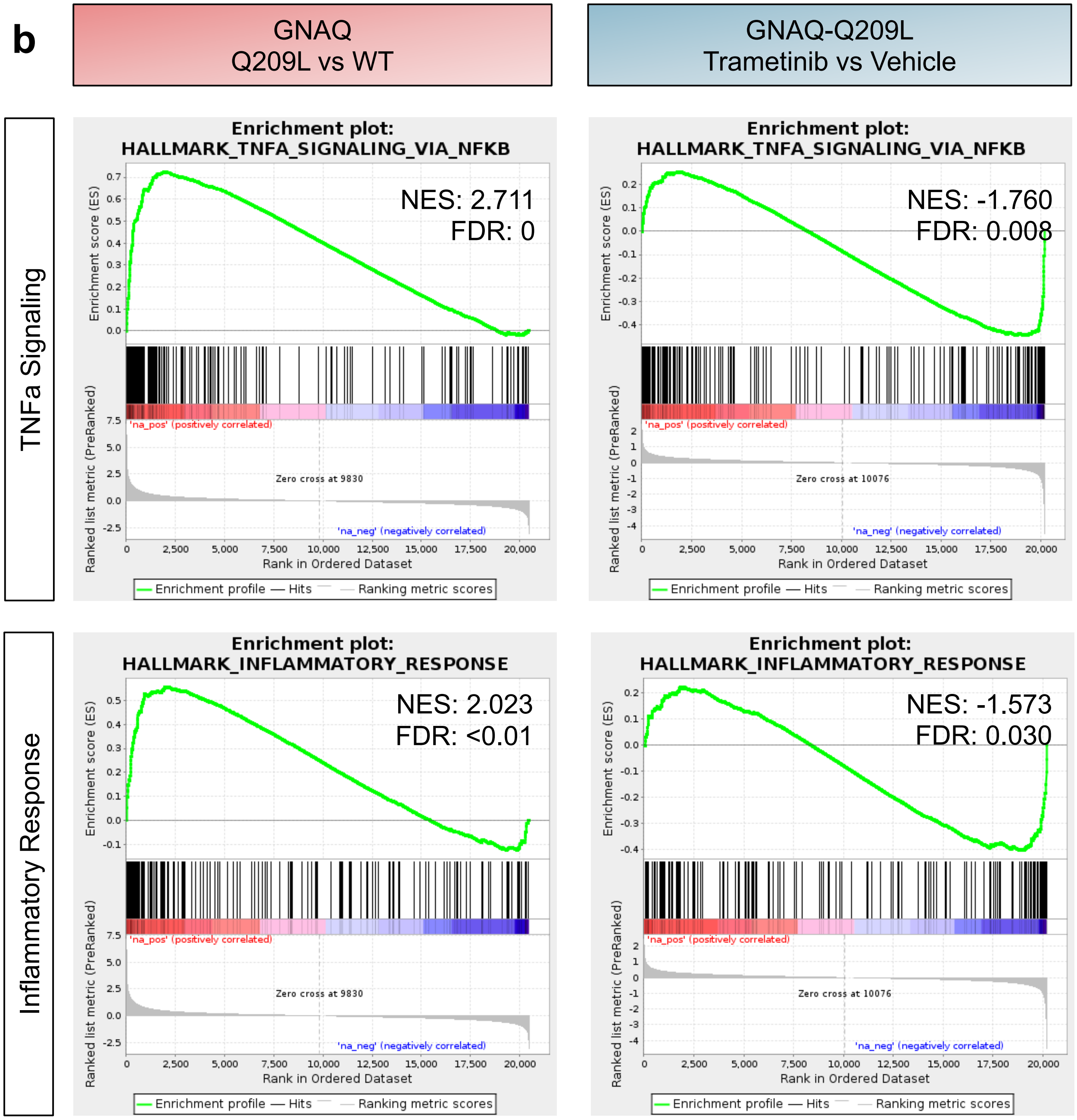

Supplementary Figure 17

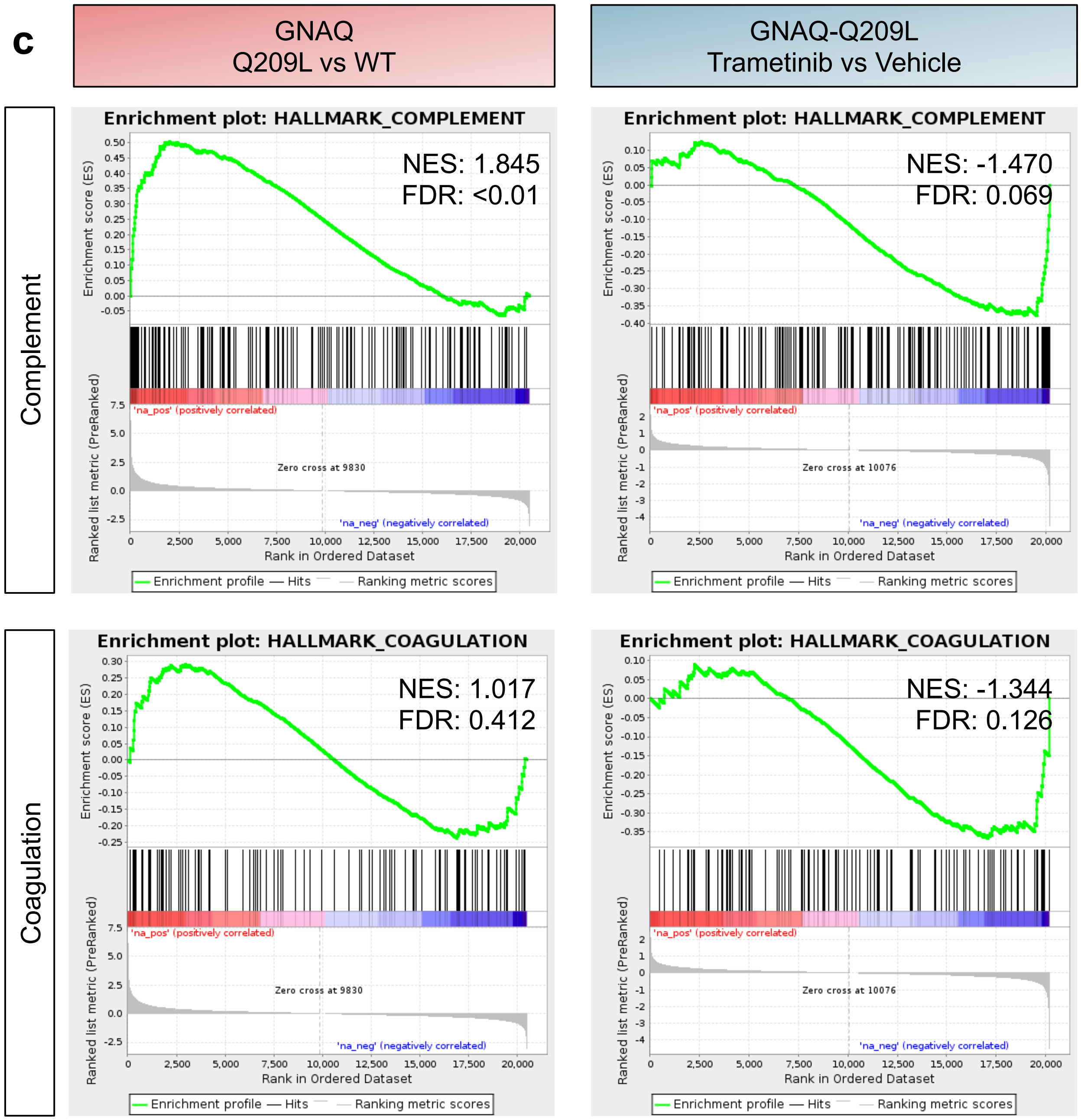

Supplementary Figure 17. Gene Set Enrichment Analysis (GSEA) in *GNAQ*^{Q209L} cells treated with Trametinib. Plots with analysis for different Gene Set Enrichment Analysis (GSEA) utilizing the Hallmark Database was performed on normalized RNA-Sequencing expression data. Pathways related to **(a)** KRAS Signaling, Angiogenesis, **(b)** TNFA Signaling via NFKB, Inflammatory response, **(c)** Complement and Coagulation are shown. (left) iEC *GNAQ*-Q209L compared iEC *GNAQ*-WT and (right) iEC *GNAQ*-Q209L treated with Trametinib compared to iEC *GNAQ*-Q209L with Vehicle. Enrichment plots are displayed with adjusted p-value (FDR) and normalized enrichment score (NES). Source data are provided in Source Data file.

Supplementary Figure 18

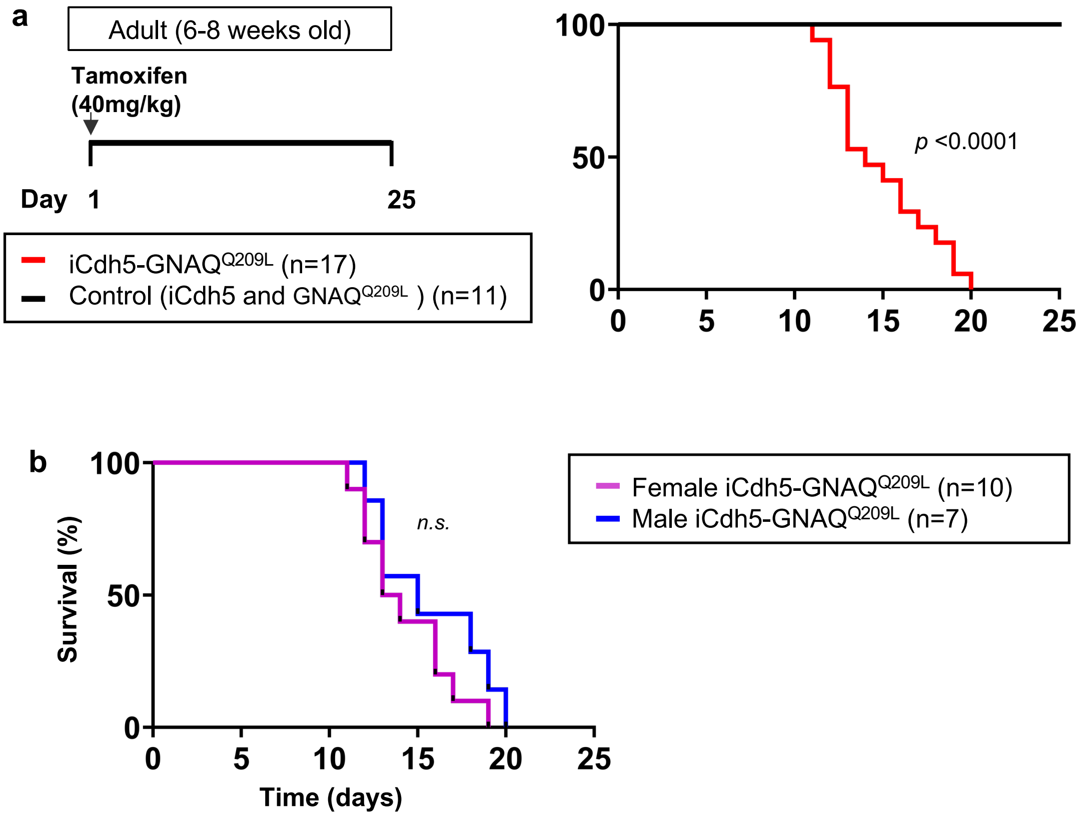

Supplementary Figure 18. Survival curves with lower dose of tamoxifen (40mg/kg). (a) Tamoxifen induction scheme and Kaplan-Meier curve comparing the survival percentage of *iCdh5-GNAQ*^{Q209L} (n=17, red) to control mice (*iCdh5*, n=5 and *GNAQ*^{Q209L}, n=6) mice**.** Gehan–Breslow–Wilcoxon test (p<0.0001). **(b)** Kaplan-Meier curve comparing the survival percentage of *iCdh5-GNAQ*^{Q209L} female (n=10, purple) to male (n=7, blue) mice. Gehan–Breslow–Wilcoxon test; n.s. for p<0.05. Source data for (a,b) are provided in Source Data file.

Supplementary Figure 19

a

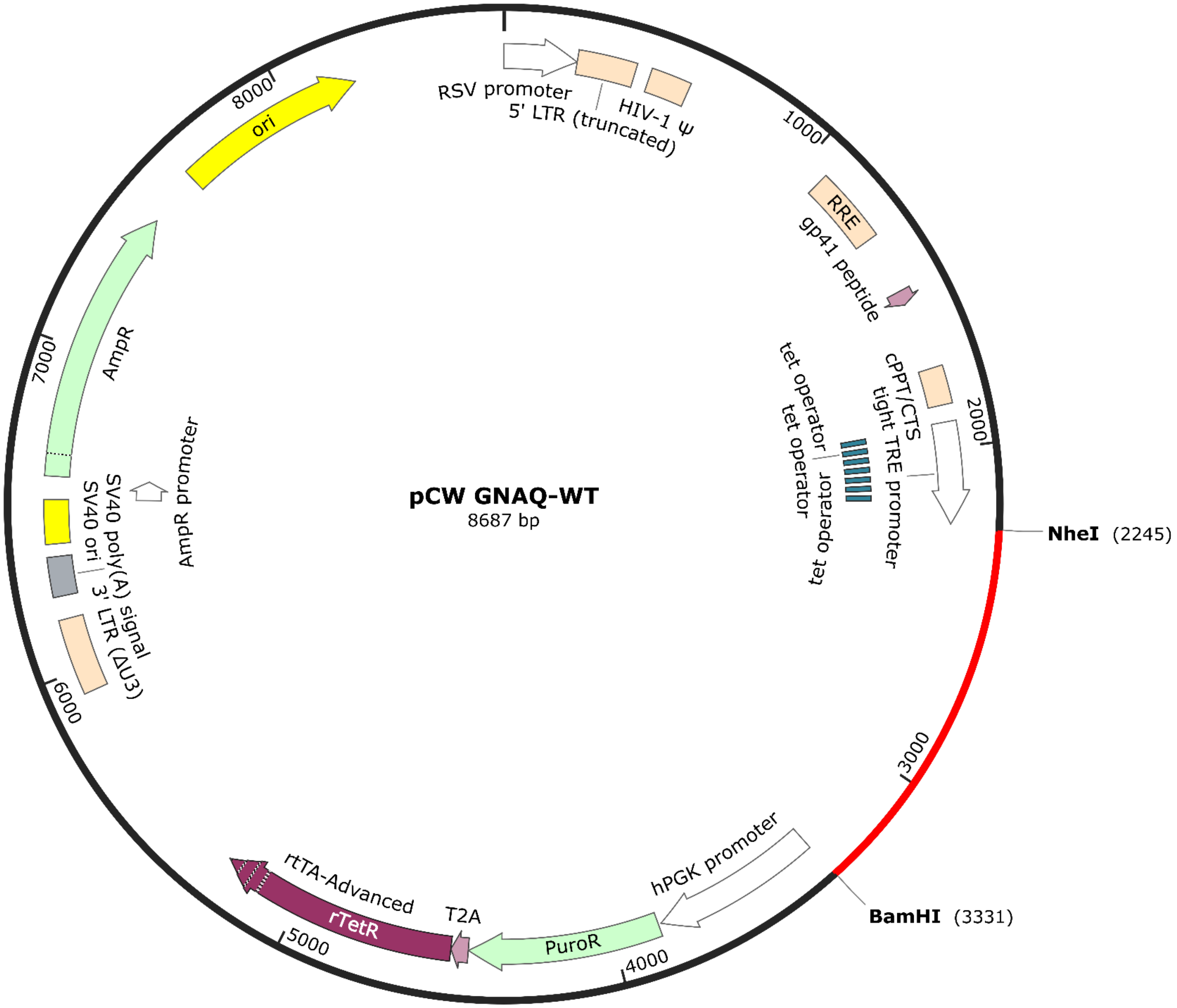

Supplementary Figure 19

b

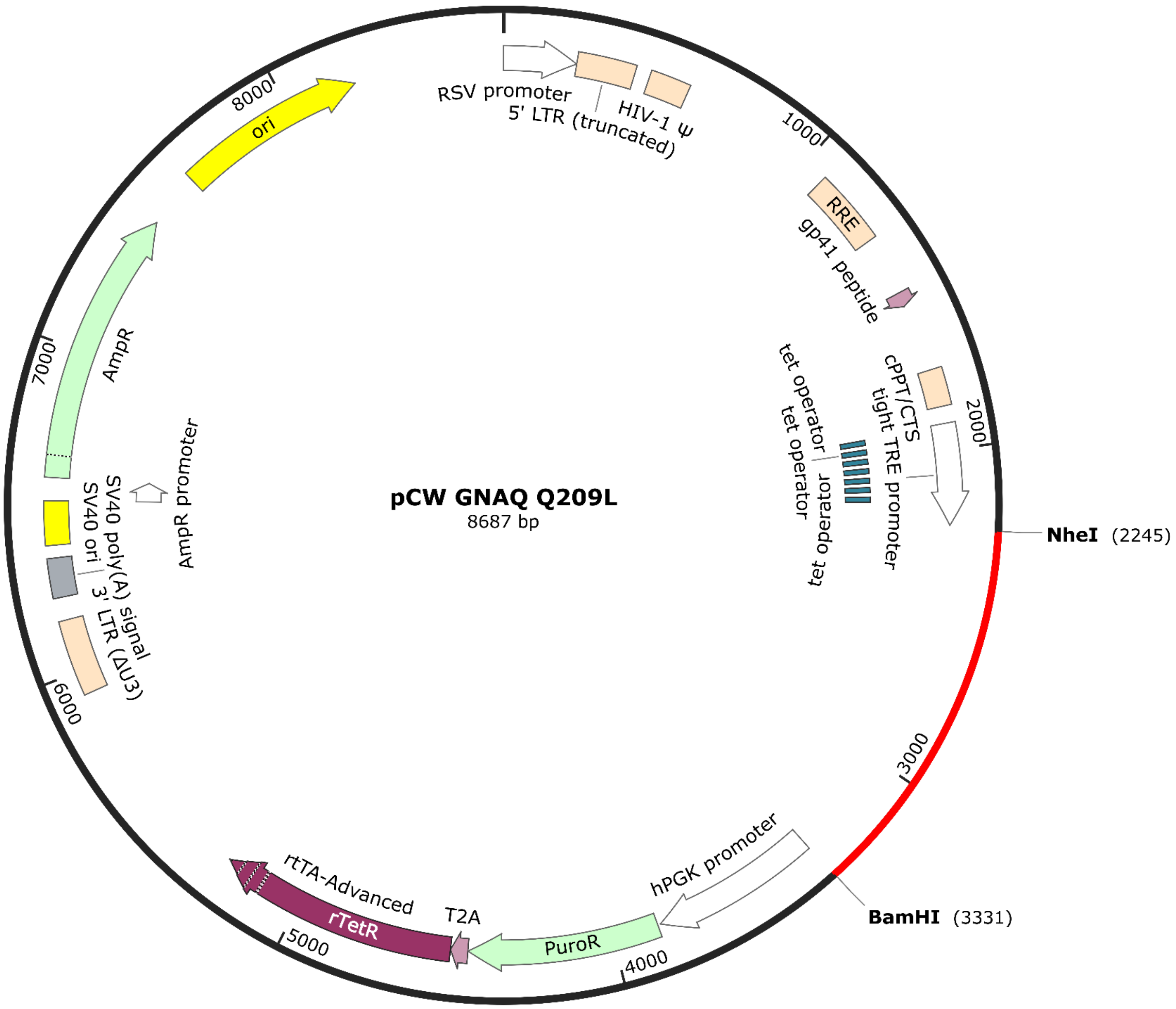

Supplementary Figure 19. Plasmid maps. Schematic of lentiviral constructs that were generated with doxycycline-inducible expression of **(a)** wild-type *GNAQ* (WT) and **(b)** mutant *GNAQ* p.Q209L. Created with SnapGene software (www.snapgene.com).
