# Supplementary material for: MEK inhibition reduced vascular tumor growth and coagulopathy in a mouse model with hyperactive GNAQ
Source: Nat Commun. 2023 Apr 6;14:1929. doi: 10.1038/s41467-023-37516-7 (PMC10079932; doi:10.1038/s41467-023-37516-7)
Supplement: Supplementary file 3 — Description to Additional Supplementary Information [file 41467_2023_37516_MOESM3_ESM.pdf]

Supplementary Movie Legends

File name: Supplementary Movie 1
Description: *iCdh5* control mouse: animated view of a three-dimensional (3D) reconstruction of whole-mount CD31-labeled (brown) subcutaneous vasculature at postnatal (P4).

File name: Supplementary Movie 2
Description: *iCdh5-GNAQ*^{Q209L} mutant mouse: animated view of a 3D reconstruction of whole-mount CD31-labeled (brown) subcutaneous vasculature at postnatal (P4).

File name: Supplementary Movie 3
Description: *iCdh5* control adult mouse: animated view of a 3D reconstruction of whole-mount intestinal *muscularis* vasculature labeled for CD31 (brown) and EdU (green).

File name: Supplementary Movie 4
Description: *iCdh5-GNAQ*^{Q209L} mutant adult mouse: animated view of a 3D reconstruction of whole-mount intestinal *muscularis* vasculature labeled for CD31 (brown) and EdU (green).

File name: Supplementary Movie 5
Description: *iCdh5* control mouse: animated view of a 3D reconstruction of whole-mount adult intestinal *muscularis* vasculature labeled for CD31 (brown) and Ter119 (white).

File name: Supplementary Movie 6
Description: *iCdh5-GNAQ*^{Q209L} mutant mouse: animated view of a 3D reconstruction of whole-mount adult intestinal *muscularis* vasculature labeled for CD31 (brown) and Ter119 (white).

File name: Supplementary Movie 7
Description: *iCdh5* control mouse**:** animated view of serial optical sections of whole-mount intestinal *muscularis* vasculature perfused and immunostained with CD31 (green), CD41 (red) and TER-119 (white).

File name: Supplementary Movie 8
Description: *iCdh5* control mouse**:** animated view of a 3D reconstruction of whole-mount intestinal *muscularis* vasculature perfused and labeled for CD31 (green), CD41 (red) and TER-119 (white).

File name: Supplementary Movie 9
Description: *iCdh5-GNAQ*^{Q209L} mutant mouse: animated view of a 3D reconstruction of whole-mount intestinal *muscularis* vasculature perfused and immunostained with CD31 (green), CD41 (red) and TER-119 (white).

File name: Supplementary Movie 10
Description: *iCdh5-GNAQ*^{Q209L} mutant mouse: animated view of serial optical sections of whole-mount intestinal *muscularis* vasculature perfused and labeled for CD31 (green), CD41 (red) and TER-119 (white).

File name: Supplementary Movie 11
Description: *iCdh5-GNAQ*^{Q209L} mouse treated with vehicle: animated view of a 3D reconstruction of whole-mount intestinal *muscularis* vasculature labeled for CD31 (brown) and EdU (green).

File name: Supplementary Movie 12
Description: *iCdh5-GNAQ*^{Q209L} mouse treated with Trametinib: animated view of a 3D reconstruction of whole-mount intestinal *muscularis* vasculature labeled for CD31 (brown) and EdU (green).
