# Supplementary material for: MEK inhibition reduced vascular tumor growth and coagulopathy in a mouse model with hyperactive GNAQ
Source: Nat Commun. 2023 Apr 6;14:1929. doi: 10.1038/s41467-023-37516-7 (PMC10079932; doi:10.1038/s41467-023-37516-7)
Supplement: Supplementary file 16 — Reporting Summary [file 41467_2023_37516_MOESM16_ESM.pdf]

nature portfolio

Corresponding author(s): Elisa Boscolo

Last updated by author(s): 03/01/2023

Reporting Summary

Nature Portfolio wishes to improve the reproducibility of the work that we publish. This form provides structure for consistency and transparency in reporting. For further information on Nature Portfolio policies, see our Editorial Policies and the Editorial Policy Checklist.

Statistics

For all statistical analyses, confirm that the following items are present in the figure legend, table legend, main text, or Methods section.

n/a	Confirmed	
☐	☒	The exact sample size (n) for each experimental group/condition, given as a discrete number and unit of measurement
☐	☒	A statement on whether measurements were taken from distinct samples or whether the same sample was measured repeatedly
☐	☒	The statistical test(s) used AND whether they are one- or two-sided<br>*Only common tests should be described solely by name; describe more complex techniques in the Methods section.*
☒	☐	A description of all covariates tested
☐	☒	A description of any assumptions or corrections, such as tests of normality and adjustment for multiple comparisons
☐	☒	A full description of the statistical parameters including central tendency (e.g. means) or other basic estimates (e.g. regression coefficient) AND variation (e.g. standard deviation) or associated estimates of uncertainty (e.g. confidence intervals)
☐	☒	For null hypothesis testing, the test statistic (e.g. F, t, r) with confidence intervals, effect sizes, degrees of freedom and P value noted<br>*Give P values as exact values whenever suitable.*
☒	☐	For Bayesian analysis, information on the choice of priors and Markov chain Monte Carlo settings
☒	☐	For hierarchical and complex designs, identification of the appropriate level for tests and full reporting of outcomes
☒	☐	Estimates of effect sizes (e.g. Cohen's d, Pearson's r), indicating how they were calculated

Our web collection on statistics for biologists contains articles on many of the points above.

Software and code

Policy information about availability of computer code

Data collection	Excel v16.67, NIS Elements AR (v.5.30.03)
Data analysis	FastQC v0.11.7, Trim Galore! v0.4.2, Cutadapt v1.9.1, STAR v2.6.1e, Sambamba v0.6.8, featureCounts v1.6.2, R package DESeq2 v1.26.0, ShinyGO v0.76.2, ggplot2 v3.3.5, pheatmap v1.0.12, GSEA v3.0, NIS Elements AR v.5.30.03, FIJI v1.54b, CellProfiler v4.2.5, Image studio v5.2, Imaris v9.8, , Angiool v0.6a, Prism v9.3.1, SnapGene Viewer v6.2, BD FlowJo version 10.8.1

For manuscripts utilizing custom algorithms or software that are central to the research but not yet described in published literature, software must be made available to editors and reviewers. We strongly encourage code deposition in a community repository (e.g. GitHub). See the Nature Portfolio guidelines for submitting code & software for further information.

Data

Policy information about availability of data

All manuscripts must include a data availability statement. This statement should provide the following information, where applicable:

- Accession codes, unique identifiers, or web links for publicly available datasets
- A description of any restrictions on data availability
- For clinical datasets or third party data, please ensure that the statement adheres to our policy

Source data are provided with this paper. The Cell profiler pipeline can be downloaded here: https://cellprofiler.org/published-pipelines. The generated RNA-Seq

data in this study has been deposited in GEO under the accession number GSE216367 (https://www.ncbi.nlm.nih.gov/geo/query/acc.cgi?acc=GSE216367). All data generated in this study are provided in the Source Data file and in Supplementary information file.

Human research participants

Policy information about studies involving human research participants and Sex and Gender in Research.

Reporting on sex and gender	Samples were obtained without identifiers and include excised tumor tissue sections and discarded neonatal foreskin for immunohistochemistry and immunofluorescent staining. We do not have information on sex of the human subject of teh tumor sample in the study. Neonatal Foreskin is routinely collected from males.
Population characteristics	Patient: affected by vascular tumor. Normal controls: foreskin tissue from 5 neonates. No exclusions. Neonatal Foreskin is routinely collected from males.
Recruitment	Vascular anomaly tissue is collected from all available specimens without regard to sex/gender or ethnicity. There are no criteria for inclusion and exclusion. No identifiers are maintained.
Ethics oversight	The study was performed in accordance with the Declaration of Helsinki, and the patient tissue sample was obtained after written informed consent. This study used samples, data, and/or services from the Discover Together Biobank at Cincinnati Children's Research Foundation.<br>All the procedures were approved by the Institutional Review Board according to ethical guidelines (Approved IRB # 2016-3878 and # 2017-3726 per institutional policies) at Cincinnati Children's Hospital Medical Center (CCHMC), with approval of the Committee on Clinical Investigation. Samples were obtained without identifiers and include excised tumor tissue sections and neonatal foreskin for immunohistochemistry and immunofluorescent staining.

Note that full information on the approval of the study protocol must also be provided in the manuscript.

Field-specific reporting

Please select the one below that is the best fit for your research. If you are not sure, read the appropriate sections before making your selection.

☒ Life sciences ☐ Behavioural & social sciences ☐ Ecological, evolutionary & environmental sciences

For a reference copy of the document with all sections, see nature.com/documents/nr-reporting-summary-flat.pdf

Life sciences study design

All studies must disclose on these points even when the disclosure is negative.

Sample size	To ensure reproducibility and statistical power sample size was estimated based on the minimum number of animals required to obtain biologically meaningful results in studies of vascular phenotype, based on scientific literature and on our laboratory experience. For in vitro studies experiments were performed at least in triplicate with 3-10 technical replicates.
Data exclusions	no exclusion
Replication	all experiments were performed with at least 3 independent replicates. All replicates were successful.
Randomization	mice were randomly assigned to vehicle or Trametinib treatment
Blinding	For animal treatment studies, mice were randomly assigned blinding was not possible as mice of defined genotypes need to be assigned to different treatment groups. Investigators were blinded to the groups during image quantification. Data which was automatically analyzed by software – analysis is inherently ignorant of experimental groups.

Reporting for specific materials, systems and methods

We require information from authors about some types of materials, experimental systems and methods used in many studies. Here, indicate whether each material, system or method listed is relevant to your study. If you are not sure if a list item applies to your research, read the appropriate section before selecting a response.

Materials & experimental systems

n/a	Involved in the study
☐	☒ Antibodies
☐	☒ Eukaryotic cell lines
☒	☐ Palaeontology and archaeology
☐	☒ Animals and other organisms
☒	☐ Clinical data
☒	☐ Dual use research of concern

Methods

n/a	Involved in the study
☒	☐ ChIP-seq
☐	☒ Flow cytometry
☒	☐ MRI-based neuroimaging

Antibodies

Antibodies used

Primaries:

rabbit anti-CD31 (Cell Signaling, #77699, 0.062µg/mL); D8V9E
rabbit anti-pERK 1/2 (Phospho-p44/42 MAPK Thr202/Tyr204, Cell signaling, #9101, 5µg/mL); (polyclonal antibody)
anti-Ki67-A488 (Cell Signaling, #11882, 2µg/mL); D3B5
anti-ERG-A647 (Abcam, #ab196149, 5µg/mL); EPR3864
UEA-I-DL649 (Vector laboratories, DL-1068-1; 20µg/mL); lectin
rat anti-mouse CD31 antibody (BD Biosciences, #550274 (clone MEC 13.3), 0.075µg/mL); MEC 13.3
A647-conjugated anti-mouse TER119 antibody (Biolegend, #116218, 5µg/mL); TER-119
CD41-PE (BD Biosciences, #558040, 1µg/mL); MWReg30
CD42b (Abcam, #ab183345, 0.3µg/ml); SP219
VE-cadherin-A647 (BD Biosciences, #561567, 0.5[illegible]g/mL); 55-7H1
rabbit anti-GNAQ (Cell Signaling, #14373, 0.05µg/mL); D5V1B
mouse anti-VE-cadherin (Santa Cruz, #sc-9989, 0.5µg/mL); F-8
mouse anti-ERK 1/2 (Cell Signaling, #4696, Technologies, 0.5µg/mL); L34F12
rabbit anti-pAKT (Ser473, Cell signaling, #4060, 0.5µg/mL); D9E
mouse anti-AKT (Cell Signaling Technologies, #2920, 0.2µg/mL); 40D4
goat anti-Angiopoietin-2 (R&D Systems, #AF-623, 1µg/mL); polyclonal
mouse anti-GAPDH (Millipore, #MAB374, 0.2µg/mL). 6C5

Secondaries for IHC IF
goat anti-rabbit A594 antibody (Invitrogen, #A32740, 8µg/mL); polyclonal
biotinylated anti-rabbit antibody (Vector laboratories, #BA-1000, 7.5µg/mL); polyclonal
biotinylated UEA-I (Vector laboratories, #B-1065, 20µg/mL); polyclonal
biotinylated goat anti-rat antibody (Vector laboratories, #BA-9401, 7.5µg/mL) polyclonal

Secondaries for WB
Donkey anti-mouse IgG-DyLightTM 680 (Invitrogen, #SA5-10170, 0.1µg/mL); polyclonal
goat anti-rabbit IgG-DyLightTM 800 (Invitrogen, #SA5-10036, 0.1µg/mL); polyclonal
donkey anti-goat IgG-DyLightTM 800 (Invitrogen, #SA5-10092, 1µg/mL) polyclonal

Flow Cytometry:

Purified rat anti-mouse TER-119 Ly-76 1.25µg/mL TER-119 #116202Biolegend
Purified rat anti-mouse/human CD11b αM integrin, Mo1, Mac1, CR3, Ly-40, C3biR, ITGAM 0.63µg/mL M1/70 #101202 Biolegend
Purified rat anti-mouse Gr1 Ly6G/Ly6C 0.63 µg/mL RB6-8C5 #108402 Biolegend
Purified rat anti-mouse B220 CD45R 1.25µg/mL RA3-6B2 #103202 Biolegend
Purified rat anti-mouse CD5 Lyt-1, Ly-1, T1, Tp67, Ly-12 0.63µg/mL 53-7.3 #100602 Biolegend
Purified rat anti-mouse CD3 T cell antigen receptor complex, T3 2.50µg/mL 17A2 #100202 Biolegend
Purified rat anti-mouse CD4 L3T4, T4 0.63µg/mL RM4-4 #100402 Biolegend
Purified rat anti-mouse CD8 T8, Lyt2, Ly-2 0.63µg/mL 53-6.7 #100702 Biolegend
PE-Cyanine5 F(ab')2-Goat Anti-Rat IgG Secondary Antibody 2.50µg/mL F(ab')2-IgG #A-10691 eBioscience
APC-eFluor780 rat anti-mouse/human cKit CD117 0.50µg/mL 2B8 #47-1171-82 eBioscience
Pacific Blue rat anti-mouse Sca-1 Ly-6A/E 1.25µg/mL D7 #108120 Biolegend
BV711 Armenian hamster anti-mouse CD48 BCM1, SLAMF2, Blast-1 2.00µg/mL HM48-1 #103439 Biolegend
PE rat anti-mouse CD150 Signaling Lymphocyte Activation Molecule (SLAM), IPO-3 0.50µg/mL TC15-12F12.2 #115904 Biolegend
Biotin rat anti-mouse Flk2 CD135, Flt3, Ly-72 5.00µg/mL A2F10 #13-1351-85 Biolegend
FITC rat anti-mouse CD34 Mucosialin 20.00µg/mL RAM34 #11-0341-85 eBioscience
PerCP-eFluor710 rat anti-mouse FcγR CD16/32 0.50µg/mL 93 #46-0161-82 eBioscience
BV605 rat anti-mouse CD41 Fibrinogen receptor, gpIIb/IIIa, integrin alpha IIb, CD41a 0.50µg/mL MWReg30 #133921 Biolegend
APC rat anti-mouse CD105 Endoglin 0.50µg/mL MJ7/18 #120413 Biolegend
BV785 rat anti-mouse CD127 IL-7 receptor α chain, IL-7Rα 2.00µg/mL A7R34 #135037 Biolegend
PE-Cyanine7 Streptavidin 0.50µg/mL #405206 Biolegend

Validation

Validation data of the antibodies which were purchased from commercial vendors are available on the manufacturer website and datasheets.

rabbit anti-CD31 (Cell Signaling, #77699, 0.062µg/mL); D8V9E
https://www.cellsignal.com/products/primary-antibodies/cd31-pecam-1-d8v9e-xp-rabbit-mab/77699
rabbit anti-pERK 1/2 (Phospho-p44/42 MAPK Thr202/Tyr204, Cell signaling, #9101, 5µg/mL); (polyclonal antibody)
https://www.cellsignal.com/products/primary-antibodies/phospho-p44-42-mapk-erk1-2-thr202-tyr204-antibody/9101
anti-Ki67-A488 (Cell Signaling, #11882, 2µg/mL); D3B5
https://www.cellsignal.com/products/antibody-conjugates/ki-67-d3b5-rabbit-mab-alexa-fluor-488-conjugate/11882
anti-ERG-A647 (Abcam, #ab196149, 5µg/mL); EPR3864
https://www.abcam.com/alexa-fluor-647-erg-antibody-epr3864-ab196149.html
UEA-I-DL649 (Vector laboratories, DL-1068-1; 20µg/mL); lectin
https://vectorlabs.com/products/glycobiology/dylight-649-ulex-europaeus-agglutinin
rat anti-mouse CD31 antibody (BD Biosciences, #550274 (clone MEC 13.3), 0.075µg/mL); MEC 13.3
https://www.bdbiosciences.com/en-us/products/reagents/flow-cytometry-reagents/research-reagents/single-color-antibodies-ruo/purified-rat-anti-mouse-cd31.550274
A647-conjugated anti-mouse TER119 antibody (Biolegend, #116218, 5µg/mL);
TER-119
https://www.biolegend.com/en-us/products/alexa-fluor-647-anti-mouse-ter-119-erythroid-cells-antibody-3277?GroupID=ImportedGROUP1
CD41-PE (BD Biosciences, #558040, 1µg/mL); MWReg30
https://www.bdbiosciences.com/en-us/products/reagents/flow-cytometry-reagents/research-reagents/single-color-antibodies-ruo/pe-rat-anti-mouse-cd41.558040
CD42b (Abcam, #ab183345, 0.3µg/ml); SP219
https://www.abcam.com/cd42b-antibody-sp219-ab183345.html
VE-cadherin-A647 (BD Biosciences, #561567, 0.5[illegible]g/mL); 55-7H1
https://www.bdbiosciences.com/en-us/products/reagents/flow-cytometry-reagents/research-reagents/single-color-antibodies-ruo/alexa-fluor-647-mouse-anti-human-cd144.561567
rabbit anti-GNAQ (Cell Signaling, #14373, 0.05µg/mL); D5V1B
https://www.cellsignal.com/products/primary-antibodies/ga-q-d5v1b-rabbit-mab/14373
mouse anti-VE-cadherin (Santa Cruz, #sc-9989, 0.5µg/mL); F-8
https://www.scbt.com/p/ve-cadherin-antibody-f-8
mouse anti-ERK 1/2 (Cell Signaling, #4696, Technologies, 0.5µg/mL); L34F12
https://www.cellsignal.com/products/primary-antibodies/p44-42-mapk-erk1-2-l34f12-mouse-mab/4696
rabbit anti-pAKT (Ser473, Cell signaling, #4060, 0.5µg/mL); D9E
https://www.cellsignal.com/products/primary-antibodies/phospho-akt-ser473-d9e-xp-rabbit-mab/4060
mouse anti-AKT (Cell Signaling Technologies, #2920, 0.2µg/mL); 40D4
https://www.cellsignal.com/products/primary-antibodies/akt-pan-40d4-mouse-mab/2920
goat anti-Angiopoietin-2 (R&D Systems, #AF-623, 1µg/mL); polyclonal
https://www.rndsystems.com/products/human-angiopoietin-2-antibody_af623
mouse anti-GAPDH (Millipore, #MAB374, 0.2µg/mL). 6C5
https://www.emdmillipore.com/US/en/product/Anti-Glyceraldehyde-3-Phosphate-Dehydrogenase-Antibody-clone-6C5,MM_NF-MAB374

Secondaries for IHC IF
goat anti-rabbit A594 antibody (Invitrogen, #A32740, 8µg/mL); polyclonal
https://www.thermofisher.com/antibody/product/Goat-anti-Rabbit-IgG-H-L-Highly-Cross-Adsorbed-Secondary-Antibody-Polyclonal/A32740
biotinylated anti-rabbit antibody (Vector laboratories, #BA-1000, 7.5µg/mL); polyclonal
https://vectorlabs.com/products/antibodies/biotinylated-goat-anti-rabbit-igg
biotinylated UEA-I (Vector laboratories, #B-1065, 20µg/mL); polyclonal
https://vectorlabs.com/products/glycobiology/biotinylated-ulex-europaeus-agglutinin
biotinylated goat anti-rat antibody (Vector laboratories, #BA-9401, 7.5µg/mL) polyclonal
https://vectorlabs.com/products/antibodies/biotinylated-goat-anti-rat-igg-mouse-adsorbed

Secondaries for WB
Donkey anti-mouse IgG-DyLightTM 680 (Invitrogen, #SA5-10170, 0.1µg/mL); polyclonal
https://www.thermofisher.com/antibody/product/Donkey-anti-Mouse-IgG-H-L-Cross-Adsorbed-Secondary-Antibody-Polyclonal/SA5-10170
goat anti-rabbit IgG-DyLightTM 800 (Invitrogen, #SA5-10036, 0.1µg/mL); polyclonal
https://www.thermofisher.com/antibody/product/Goat-anti-Rabbit-IgG-H-L-Cross-Adsorbed-Secondary-Antibody-Polyclonal/SA5-10036
donkey anti-goat IgG-DyLightTM 800 (Invitrogen, #SA5-10092, 1µg/mL) polyclonal
https://www.thermofisher.com/antibody/product/Donkey-anti-Goat-IgG-H-L-Cross-Adsorbed-Secondary-Antibody-Polyclonal/SA5-10092

Flow Cytometry:

Purified rat anti-mouse TER-119 Ly-76 1.25µg/mL TER-119 #116202Biolegend
https://www.biolegend.com/de-at/products/purified-anti-mouse-ter-119-erythroid-cells-antibody-1869?GroupID=ImportedGROUP1
Purified rat anti-mouse/human CD11b αM integrin, Mo1, Mac1, CR3, Ly-40, C3biR, ITGAM 0.63µg/mL M1/70 #101202 Biolegend
https://www.biolegend.com/en-us/products/purified-anti-mouse-human-cd11b-antibody-351?GroupID=BLG10660
Purified rat anti-mouse Gr1 Ly6G/Ly6C 0.63 µg/mL RB6-8C5 #108402 Biolegend
https://www.biolegend.com/de-de/products/purified-anti-mouse-ly-6g-ly-6c-gr-1-antibody-462
Purified rat anti-mouse B220 CD45R 1.25µg/mL RA3-6B2 #103202 Biolegend
https://www.biolegend.com/en-us/search-results/purified-anti-mouse-human-cd45r-b220-antibody-449
Purified rat anti-mouse CD5 Lyt-1, Ly-1, T1, Tp67, Ly-12 0.63µg/mL 53-7.3 #100602 Biolegend
https://www.biolegend.com/it-it/search-results/purified-anti-mouse-cd5-antibody-162
Purified rat anti-mouse CD3 T cell antigen receptor complex, T3 2.50µg/mL 17A2 #100202 Biolegend

https://www.biolegend.com/fr-ch/products/purified-anti-mouse-cd3-antibody-48
Purified rat anti-mouse CD4 L3T4, T4 0.63µg/mL RM4-4 #100402 Biolegend
https://www.biolegend.com/en-us/products/purified-anti-mouse-cd4-14184
Purified rat anti-mouse CD8 T8, Lyt2, Ly-2 0.63µg/mL 53-6.7 #100702 Biolegend
https://www.biolegend.com/en-us/search-results/purified-anti-mouse-cd8a-antibody-157?GroupID=BLG2559
PE-Cyanine5 F(ab')2-Goat Anti-Rat IgG Secondary Antibody 2.50µg/mL F(ab')2-IgG #A-10691 eBioscience
https://www.thermofisher.com/antibody/product/Goat-anti-Rat-IgG-H-L-Secondary-Antibody-Polyclonal/A-10691
APC-eFluor780 rat anti-mouse/human cKit CD117 0.50µg/mL 2B8 #47-1171-82 eBioscience
https://www.thermofisher.com/antibody/product/CD117-c-Kit-Antibody-clone-2B8-Monoclonal/47-1171-82
Pacific Blue rat anti-mouse Sca-1 Ly-6A/E 1.25µg/mL D7 #108120 Biolegend
https://www.biolegend.com/en-us/products/pacific-blue-anti-mouse-ly-6a-e-sca-1-antibody-3140?GroupID=BLG2524
BV711 Armenian hamster anti-mouse CD48 BCM1, SLAMF2, Blast-1 2.00µg/mL HM48-1 #103439 Biolegend
https://www.biolegend.com/en-us/products/brilliant-violet-711-anti-mouse-cd48-antibody-14068?GroupID=BLG6848
PE rat anti-mouse CD150 Signaling Lymphocyte Activation Molecule (SLAM), IPO-3 0.50µg/mL TC15-12F12.2 #115904 Biolegend
https://www.biolegend.com/en-us/products/pe-anti-mouse-cd150-slam-antibody-1369?GroupID=BLG10572
Biotin rat anti-mouse Flk2 CD135, Flt3, Ly-72 5.00µg/mL A2F10 #13-1351-85 Biolegend
https://www.biolegend.com/en-us/products/biotin-anti-mouse-cd135-antibody-6270
FITC rat anti-mouse CD34 Mucosialin 20.00µg/mL RAM34 #11-0341-85 eBioscience
https://www.thermofisher.com/antibody/product/CD34-Antibody-clone-RAM34-Monoclonal/11-0341-82
PerCP-eFluor710 rat anti-mouse FcγR CD16/32 0.50µg/mL 93 #46-0161-82 eBioscience
https://www.thermofisher.com/antibody/product/CD16-CD32-Antibody-clone-93-Monoclonal/46-0161-82
BV605 rat anti-mouse CD41 Fibrinogen receptor, gpIIb/IIIa, integrin alpha IIb, CD41a 0.50µg/mL MWReg30 #133921 Biolegend
https://www.biolegend.com/en-us/products/brilliant-violet-605-anti-mouse-cd41-antibody-9927?GroupID=BLG10424
APC rat anti-mouse CD105 Endoglin 0.50µg/mL MJ7/18 #120413 Biolegend
https://www.biolegend.com/en-us/products/apc-anti-mouse-cd105-antibody-6519?GroupID=BLG10724
BV785 rat anti-mouse CD127 IL-7 receptor α chain, IL-7Rα 2.00µg/mL A7R34 #135037 Biolegend
https://www.biolegend.com/en-us/products/brilliant-violet-785-anti-mouse-cd127-il-7ralpha-antibody-10803
PE-Cyanine7 Streptavidin 0.50µg/mL #405206 Biolegend
https://www.biolegend.com/nl-nl/products/pe-cyanine7-streptavidin-1477

Eukaryotic cell lines

Policy information about cell lines and Sex and Gender in Research

Cell line source(s): normal human cord blood endothelial colony forming cells (ECFC) commercially available (StemBioSys)

Authentication: We utilized normal human cord blood endothelial colony forming cells (ECFC) commercially available (StemBioSys), these cells were isolated from normal donors. We have extensively characterized these cells for expression of endothelial specific marker CD31, VE-Cadherin, vWF, and do not express lymphatic markers (Prox1 and D2-40), or aSMA. These ECFC have also been tested for formation of vascular lumens in fibrin gel assay, attesting their endothelial phenotypic properties. Doxycycline-inducible GNAQ-Q209L and GNAQ-WT constructs were generated in the PI's laboratory. We have confirmed that these cells overexpress GNAQ and only the mutant GNAq-Q209L show hyperactivation of the MAPK pathway (p-ERK).

Mycoplasma contamination: All the cell lines used tested negative for mycoplasma, we perform mycoplasma tests every 3-6 months.

Commonly misidentified lines (See ICLAC register): No commonly misidentified cell lines were used in this study.

Animals and other research organisms

Policy information about studies involving animals; ARRIVE guidelines recommended for reporting animal research, and Sex and Gender in Research

Laboratory animals: mouse, C57BL/6, age E8.5-14 weeks old, female and male mice were used.
Mice strains used in these studies are are the following: Rosa26-floxed stop-GNAQQ209L which was obtained by MTA from Dr. Catherine Van Raamsdonk , Cdh5-icreERT2 obtained by MTA from Dr. Ralf Adams, Pdgfb-CreERT2 , mT/mG (Jax No.00757) 6. C57/black6 mice (WT) are purchase from JAX for breeding transgenic colonies to WT.
Procedures: 1-Transgene expression is activated by injecting adult mice, with tamoxifen (ip), 75mg/kg daily for 2 days consecutively or 40mg/kg once. 2-In these models we analyzed the development of abnormal vasculature in the subcutaneous tissue and intestine as endpoint. 3-Vessel permeability was assessed by intradermal injection of vehicle (PBS/saline) or VEGF-A (200ng/mL) and Evans blue injected via tail vein or retro-orbital (under anesthesia), let it circulate for 10 minutes and then mice will be euthanized for analysis. 4-Cell proliferation assessed by a single intraperitoneal injection of EdU 100mg/kg, mice sacrificed after 24 hours for organ collection. 5-Drug treatment (Trametinib) performed via oral gavage, daily at 2mg/kg dose. 6- Blood will collected via vena cava at endpoint to analyze CBC and KMP-related parameters (plasma D-dimer, fibrinogen).
Mice were monitored and inspected twice/week and weight recorded weekly. Mice develop vascular malformations/tumors and are to be euthanized if vascular lesions are visible and grow in size to exceed 400mm^3. If mice appear to be inactive, lethargic, with breathing difficulties, wounds get infected or losing weight (>20%), the veterinarian will be consulted and if deemed the best course, the animals will be euthanized.

Wild animals: No wild animals were used in this study.

Reporting on sex: Both female and male mice at defined ages (6-12 weeks) are used to ensure consistency. For survival studies of iCdh5-GNAQQ209L

Reporting on sex	mice (75mg/kg or 40mg/kg of tamoxifen schemes) female and male mice data was analyzed separately and did not show significant differences between the two groups. Therefore, in subsequent studies, data from female and male mice were pooled together.
Field-collected samples	No field collected samples were used in this study.
Ethics oversight	All animal procedures described below have been reviewed and approved by the CCHMC Institutional Animal Care and Use Committee (Protocol number IACUC 2020-0039).

Note that full information on the approval of the study protocol must also be provided in the manuscript.

Flow Cytometry

Plots

Confirm that:

☒ The axis labels state the marker and fluorochrome used (e.g. CD4-FITC).

☒ The axis scales are clearly visible. Include numbers along axes only for bottom left plot of group (a 'group' is an analysis of identical markers).

☒ All plots are contour plots with outliers or pseudocolor plots.

☒ A numerical value for number of cells or percentage (with statistics) is provided.

Methodology

Sample preparation	Each bone marrow (BM) sample was flushed from medullary cavity of 2 femurs and 2 tibia and into Hank's Buffered Salt Solution (HBSS) supplemented with 2% heat-inactivated fetal bovine serum (FBS) using a needle. Red blood cells were lysed for 1 minute on ice in 150mM NH4CL and 10mM KHCO3 lysis buffer, then washed and centrifuged. Single cell suspensions were obtained by suspending the samples in HBSS – 2% FBS and then filtering through 50 µm mesh. Antibody labeling for hematopoietic stem cell populations was performed on 8x106 unfractionated BM cells. After diluting antibody cocktails in HBSS – 2% FBS, cells were stained on ice in the dark for 20 to 30 minutes, except for initial lineage staining which incubated for 40 to 45 minutes, and Zombie NIR Cell Viability staining which incubated at room temperature for 4 to 5 minutes. Final samples for spectral flow cytometry analysis were filtered again using 50 µm mesh and then suspended in HBSS – 2% FBS.
Instrument	Cytek Aurora
Software	Cytek Aurora; software Cytek SpectroFlo version 3.0.3.<br>BD FlowJo version 10.8.1
Cell population abundance	HSC and progenitor cell populations were enriched by first gating on populations that were negative for lineage-specific markers, and then positively selecting for expression of stem cell antigen 1 (Sca1) and the stem cell factor receptor (c-Kit). The resulting LSK population was approximately 5 to 10% of total BM as determined by flow cytometry. HSC and progenitor cell populations were then refined using signaling lymphocyte activation molecule (SLAM) markers, CD150 and CD48. Final HSCs and progenitors had an abundancy of approximately 0.01-0.04%.
Gating strategy	Doublets and multiplets were first excluded based on cell size using the forward scatter (FSC) width and the FSC area parameters. Debris was excluded and cells of interest were identified based on FSC area and cell granularity, or side scatter (SSC) area. To increase the resolution of the single cells, a secondary doublet exclusion was performed using SSC area and SSC height. Live cells were gated using Zombie NIR Cell Viability Dye. Positive and negative populations throughout staining protocols were validated with unstained and fluorescence minus one (FMO) controls as described previously (Solomon et al., 2020). Single stained reference controls were prepared using UltraComp ebeads and the peak emission spectra of each fluorophore was obtained through the Ordinary Least Squares Linear Unmixing algorithm of the spectral flow cytometer. For the manuscript, located in the supplementary figures, we have supplied one example of the gating strategy used to compare the hematopoietic stem and progenitor cell populations of the iEC-GNAQQ209L mutant and control mice.

☒ Tick this box to confirm that a figure exemplifying the gating strategy is provided in the Supplementary Information.
